# One-Pot Approach Towards Peptoids Synthesis Using 1,4-Dithiane-2,5-Diol via Multicomponent Approach and DFT-Based Computational Analysis

**DOI:** 10.3390/molecules30112340

**Published:** 2025-05-27

**Authors:** Musrat Shaheen, Akbar Ali

**Affiliations:** Department of Chemistry, Government College University Faisalabad, Faisalabad 38000, Pakistan

**Keywords:** Ugi-4CR, peptoids, DFT study, nonlinear optical properties, natural bond order

## Abstract

Peptoids are peptidomimetics in which the side chain is attached to the nitrogen of the amide group rather than the *α*-carbon. This alteration in the backbone structure is highly valued because it endows beneficial properties, including enhanced resistance to proteolysis, greater immunogenicity, improved biostability, and superior bioavailability. In this current study, we focused on the Ugi-4CR-based one-pot synthesis of peptoids using 1,4-dithiane-2,5-diol as the carbonyl component together with amine, carboxylic acid, and isocyanides. Four new peptoids—**5a**, **5b**, **5c**, and **5d**—were designed and efficiently prepared in good chemical yields and were subjected to DFT investigations for their electronic behavior. These compounds have free OH, SH, and terminal triple bonds for further chemistry. In a computational analysis, the spectral data of compounds **5a**–**5d** were juxtaposed with calculated spectral values derived from the B3LYP/6-311G(d,p) level. The electronic excitation and orbital contributions of **5a**–**5d** were predicted using TD-DFT calculations. A natural bond order (NBO) analysis was utilized to investigate the electronic transition of newly synthesized peptoids, focusing on their charge distribution patterns. Furthermore, MEP and NPA analyses were conducted to predict charge distribution in these compounds. The reactivity and stability of the targeted peptoids were evaluated by global reactivity descriptors, which were determined with frontier molecular orbital analysis. The DFT results revealed that compound **5c** displayed marginally higher reactivity compared to **5a**, **5b**, and **5d**, possibly due to its extended conjugation.

## 1. Introduction

**Figure 1:**
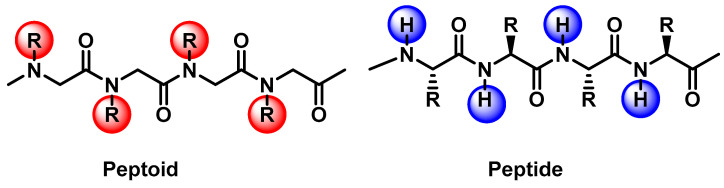
Representative structures of the peptide and peptoid backbones.

Peptides are *N*-substituted glycine derivatives that prevent proteolysis by relocating the side chain from the *α*-carbon to the amide nitrogen (Figure 1) [1,2]. Hence, peptoids can specifically be designed for various purposes in terms of biological activities, including antioxidant, antibacterial, antifouling, antifungal, and anticancer, as well as optoelectronic properties. In the chemical structure of peptides, each amino acid is unique, as it has different side-chains and can be used for various applications [3,4]. However, they are less resistant to proteolysis in biological systems and can have less biostability and low bioavailability. Peptoids, on the other hand, are peptidomimetics that can be easily and inexpensively synthesized, and they can overcome these obstacles for applications in various biological fields [5,6,7].

**Figure 2:**
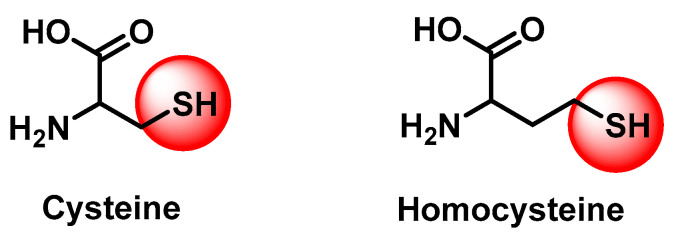
Structures of SH-containing amino acids.

The Ugi four-component reaction is an excellent one-pot approach (utilizing amine, isocyanide, carbonyl, and carboxylic acid groups) for the synthesis of peptoids in a single step with a diverse spectrum of applications [6,8,9]. Sulfur-containing peptides are of high interest in peptide therapeutics due to their unique capabilities [7,10]. These compounds play a vital role in biological systems, enhancing stability and pharmacokinetic features, such as prolonged half-life and receptor-specific interactions [11]. Cysteine and homocysteine (Figure 2) are unique sulfur-containing amino acids characterized by the redox-active sulfhydryl (-SH) group in their side-chains, exhibiting high reactivity in the biological functions [12].

**Figure 3:**
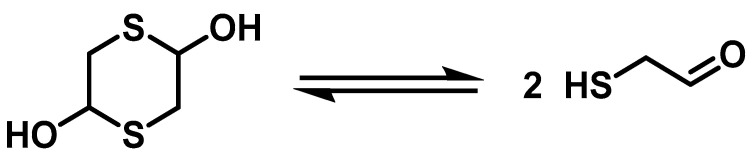
Interconversion of 1,4-dithiane-2,5-diol and 2-mercaptoacetaldehyde.

1,4-Dithiane-2,5-diol serves as a source of sulfur for the in situ production of 2-mercaptoacetaldehyde, a flexible two-carbon synthon with both electrophilic and nucleophilic reactive sites. 2-mercaptoacetaldehyde is extensively used as a platform for synthesizing sulfur-containing compounds and can act as the aldehyde component in Ugi 4-CR reactions for peptoid synthesis. Thus, 1,4-dithiane-2,5-diol is a flexible dimer of 2-mercaptoacetaldehydes that contain sulfur atoms (Figure 3).

Recently, the DFT-based exploration of peptoids for critical insight has gained considerable attention from the scientific community due to peptoids’ structural versatility, tunable bioactivity, and emerging applications in material science and catalysis. As peptoids present unique folding patterns owing to the absence of hydrogen bonding in their backbones, DFT calculation helps to predict the stable conformers and energetics [13]. Similarly, DFT calculations have been used to forecast the electronic properties and reactivity of peptoids by calculating the charge distribution and orbital interactions, as well as reaction pathways such as peptoid–metal binding for catalysis [14]. Moreover, computer-assisted calculation can be employed to ascertain certain other features of peptoids, like for the quantification of non-covalent interactions (π-stacking and Van der Waals and hydrophobic effects in peptoid self-assembly) and bioactivity in terms of peptoid–protein binding, including enzyme inhibition and pharmacokinetics such as solubility [15,16].

The one-pot production of such types of compounds is well known in various domains such as pharmaceuticals, agrochemicals, and functional materials, including optoelectronic applications. Importantly, these generated building blocks can be further diversified to produce even more interesting molecules for different purposes, such as generating metal–organic frameworks known as MOFs and heavy metal sensors due to the presence of free OH and SH functionalities. Furthermore, these types of compounds could be utilized in addressing contemporary scientific and industrial challenges as well and could have a leading role in the field of bioconjugation due to the presence of free terminal alkyne functional groups.

Keeping in mind the importance of the computer-assisted calculation of peptoids, herein, we present our findings regarding the synthesis of four new SH, OH, and terminal triple bonds incorporated in peptoid molecules using 1,4-dithiane-2,5-diol as carbonyl components together with amine, carboxylic acid, and isocynide in a one-pot approach that uses Ugi four-component reactions and DFT analysis to estimate the electronic, structural, and topological characteristics of these peptoids [17,18,19,20].

## 2. Results and Discussion

### 2.1. Computational Study

**Figure 4:**
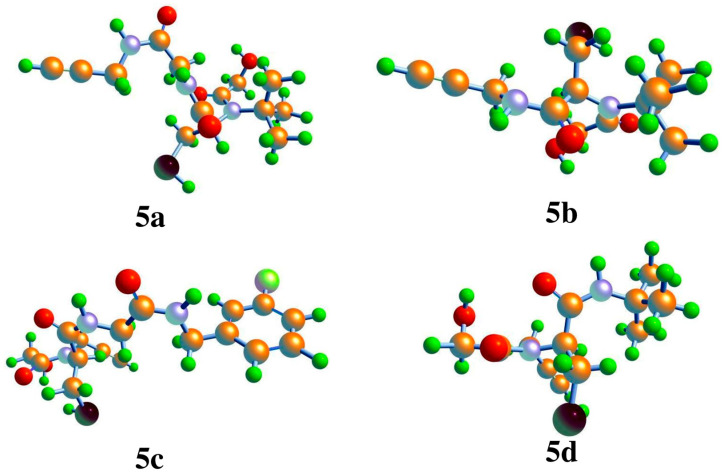
Calculated geometries of the newly accomplished compounds **5a**–**5d**.

This section presents a computational analysis of the newly synthesized compounds (**5a**–**5d**) using DFT calculations. The Lee–Yang–Parr B3LYP/6-311G (d,p) basis set [21] was employed to perform these theoretical assessments. The PCM (polarizable continuum models) [22] was employed to investigate the solvent effects. The selected basis set, 6-311G(d,p), includes polarization functions to address electronic cloud distortion, thereby enhancing the accuracy of the calculated molecular properties. Frequency calculations were performed at the same level of theory (B3LYP/6-311G(d,p)) to confirm that the optimized geometries correspond to true minima (indicated by the absence of imaginary frequencies) and to incorporate zero-point energy (ZPE) corrections. The ZPE corrections for the synthesized compounds **5a**–**5d** were 0.3703, 0.3139, 0.3623, and 0.3623 Hartree, respectively [23]. This study generated molecular electrostatic potential (MEP) maps and frontier molecular orbital (FMO) distributions. Additionally, various properties were determined, including the energies of the highest occupied molecular orbital and the lowest unoccupied molecular orbital, along with the energy gaps between HOMO and LUMO. Figure 4 displays the optimized configurations of compounds **5a**–**5d**. The effect of solvent on the bond angles was marginal. Similarly, the solvent had a minor effect on the bond lengths and bond angles (Table 1), as reflected in the computed values [24,25].

### 2.2. Vibrational Spectroscopy

The FTIR bands observed were used to calculate the relative intensities for compounds **5a**–**5d**. These calculations were performed using the DFT/B3LYP/6-311G(d,p) [26] computational technique to estimate the vibrational modes and their intensities (Tables S1–S4). To correct systematic errors arising from basis set limitations, insufficient electron correlation, and vibrational anharmonicity, the theoretical DFT values were adjusted using a scale factor of 0.97 to match the experimental results [27,28,29]. The spectral studies revealed the following vibrational modes:

#### 2.2.1. C–H Vibrations

Aromatic C–H vibrations were generally found within the range of 3000–3100 cm^−1^ [30,31]. For compound **5a**, the DFT-calculated C–H vibrations were found at 3105 cm^−1^ (Table S1), which aligned with the experimental value of 3063 cm^−1^ (Figure S13). Similarly, in compound **5b**, the DFT-calculated C–H vibrations were identified at 3074 cm^−1^ (Table S2), corresponding to the experimental values of 3074 cm^−1^ (Figure S14). In compound **5c**, the C–H vibrations were identified at 3148 cm^−1^ (Table S3), with the DFT value correlating with the experimental measurement of 3058 cm^−1^ (Figure S15). For compound **5d**, the C–H modes were found at 3074 cm^−1^ (Table S4), with the DFT-calculated value closely matching the experimental value of 3062 cm^−1^ (Figure S16).

#### 2.2.2. O-H Vibrations

The O–H vibrations were detected within the range of 3750–3100 cm^−1^ [32,33]. For compounds **5a**–**5d**, DFT calculations revealed O–H vibrations at 3654, 3632, 3658, and 3621 cm^−1^, respectively. These values align with the experimental measurements of 3751, 3780, 3788, and 3795 cm^−1^ (Figures S13–S16).

#### 2.2.3. N-H Vibrations

N–H vibrations were detected within the 3300–3600 cm^−1^ range [34,35]. In compound **5a**, the amide N–H vibration appeared at a computed value of 3557 cm^−1^ (Table S1), closely matching the experimental value of 3345 cm^−1^ (Figure S13). For compound **5b**, the amide N–H vibration appeared at a computed value of 3539 cm^−1^ (Table S2), aligning with the experimental result of 3287 cm^−1^ (Figure S14). For compound **5c**, the amide N-H vibrations were observed at 3538 cm^−1^ (DFT value, Table S3), which correlates with the experimental value of 3524 cm^−1^ (Figure S15). For compound **5d**, the amide N-H vibration was found at a computed value of 3508 cm^−1^ (Table S4), correlating with 3274 cm^−1^ (Figure S16) in the experimental spectrum.

#### 2.2.4. Carbon–Sulfur (C–S) Vibrations

The carbon–sulfur (C–S) bond typically exhibits vibrations within the range of 600–700 cm^−1^ [36]. For compounds **5a**–**5d**, the carbon–sulfur stretching modes were calculated using DFT and were 623, 610, 690, and 607 cm^−1^, respectively (Tables S1–S4). These computational results align with the experimental values of 627, 602, 736, and 721 cm^−1^ (Figures S13–S16), respectively.

#### 2.2.5. Carbon–Nitrogen (C–N) Vibrations

The C–N bond exhibits vibrations within the 1500–500 cm^−1^ range [10,37]. For compound **5a**, carbon–nitrogen vibration modes were detected in the IR spectrum at 1455–822 cm^−1^ using DFT (Table S1), aligning with the experimental value in the FTIR spectrum of 1457–846 cm^−1^ (Figure S13). C–N–C vibration modes were found at 995–782 cm^−1^ via DFT (Table S2), corresponding to the experimental measurement of 959–782 cm^−1^ (Figure S13). For compound **5b**, C–N modes were found at 1530–583 cm^−1^ through DFT (Table S2), corresponding to the experimental range of 1520–535 cm^−1^ (Figure S14). Compound **5c** exhibited C–N stretching vibrations at 1510–1095 cm^−1^ (Table S3) in the FTIR spectrum, correlating with the experimental measurement of 1522–1028 cm^−1^ (Figure S15). For compound **5d**, carbon–nitrogen vibrations appeared at 1468–1203 cm^−1^ using DFT values (Table S4), correlating with the experimental value of 1507–916 cm^−1^ (Figure S16).

#### 2.2.6. C=O Vibrations

C=O stretching vibrations are typically observed in the spectral range of 1850–1600 cm^−1^ [38]. For compound **5a**, DFT calculations revealed the conjugated C=O (amide) vibration at 1755–1683cm^−1^, respectively (DFT) (Table S1), which aligns with the experimental result showing a shift of 1695 cm^−1^ (Figure S13). Compound **5b** exhibited a conjugated C=O (amide) vibration at 1713–1699 cm^−1^ (Table S2) according to DFT calculations, consistent with the experimental range of 1830 cm^−1^ (Figure S14). DFT analysis of compound **5c** showed the conjugated C=O (amide) vibration at 1758 cm^−1^ (Table S3), corresponding with the experimental range of 1771 cm^−1^ (Figure S15). For compound **5d**, DFT calculations indicated the conjugated C=O vibration (amide) at 1722–1700 cm^−1^ (Table S4), which matches the experimental measurement of 1698 cm^−1^ (Figure S16).

#### 2.2.7. C=C Stretching Vibrations

The stretching vibrations of the C=C bond were typically found within the range of 1660 to 1600 cm^−1^, while for aromatic systems, they were between 1600 and 1475 cm^−1^ [39]. In the case of compound **5c**, DFT calculations revealed C=C (aromatic) stretching vibrations at 1702 cm^−1^ (Table S3). These computational results align with an experimental measurement of 1717 cm^−1^ (Figure S15).

#### 2.2.8. C≡C Stretching Vibrations

The C≡C bond stretching vibrations were typically found within the range of 2000–2300 cm^−1^ [40]. In the titled compounds **5a**–**5d**, the C≡C stretching vibrations were found at 2232, 2232, 2252, and 2223 cm^−1^, respectively (Tables S1–S4), using DFT calculations, which closely matched the experimental range of 2095–2137 cm^−1^ (Figures S13–S16).

### 2.3. NBO Analysis

Natural bond orbital analysis serves as an effective approach for investigating electronic distribution within the atoms and among the bonds within the molecule. Furthermore, it establishes a valuable framework for studying conjugative interactions and charge transfer within the molecular system [41,42,43]. This study revealed results concerning specific electron donor and electron acceptor orbitals, along with the stabilization energy derived from their interaction, which was calculated using second-order perturbation theory. The ***E***^(**2**)^ value (Equation (1)) was directly linked to the magnitude of electron–donor interactions and the degree of conjugation throughout the system. The stabilizing interaction between the donor and acceptor was linked to the shift in electron density from filled Lewis-type NBO orbitals, including bonds or lone pairs, to non-Lewis NBO orbitals, which are typically anti-bonding or Rydberg and formally unoccupied [44]. To explore the changes at the molecular level in hybridization and electron distribution, NBO analysis was conducted via the DFT/B3LYP/6-31G(d,p) theoretical method. The molecular interaction occurred due to the overlap between the σ (C–C)→σ*(C–C) bond orbitals. This overlap enabled ICT (intramolecular charge transfer), which contributed to the stabilization of the system.

(1) $$E^{\left(2\right)}=q_{i}\frac{\;{\left(F_{i,\;j}\right)}^{2}}{{Ɛ}_{j}-{Ɛ}_{i}}$$

***E*^(2)^** signifies the stabilization energy, also referred to as hyper-conjugative interaction energy. The energy gap between the donor and acceptor NBO orbitals *i* and *j* is expressed as Ɛ_(j)_ − Ɛ_(i)_. F(_i,j_)^2^ represents the Fock matrix element between i and j NBO orbitals, *qi* denotes the population of the donor orbital, and Ɛj and Ɛi correspond to the energies of NBO [45]. Tables S5–S8 contains a more comprehensive description of the NBO analysis for compounds **5a**–**5d**. The compounds under examination predominantly display six categories of electronic transitions. Among these, the most common transitions observed are π→π*, LP→π*, and LP→σ*, whereas σ→σ* transitions are the least frequent. An examination of orbital stabilization energies in compound **5a** revealed the most prominent transitions to be LP→π* interactions. These interactions arose from resonance between electron-donating LP (1)N18 and LP (1)N4 and electron-accepting π*C17-O19 and π*C11-O15 anti-bonding orbitals, with corresponding stabilization energies of 69.24 and 1.4 kjmol^−1^, respectively. Additionally, significant hyperconjugative LP→σ* transitions were identified between electron-donating LP (2)O5 and LP (2)O15 and electron-accepting σ*C3-N4 and σ*C10-H34 anti-bonding orbitals, resulting in stabilization energies of 23.31 and 0.51 kjmol^−1^, respectively. Moreover, significant intramolecular hyperconjugative π→π* transitions were observed through orbital overlap between πC17-O19→π*C17-O19, πC11-O15→π*C11-O15, and πC3-O5→π*C3-O5, with corresponding stabilization energies of 1.33, 1.28, and 0.92 kjmol^−1^, respectively (Table S5). In the studied compound **5b**, several electronic transitions were identified. The π→π* transitions include πC11-O13→π*C11-O13, with a stabilization energy of 1.05 kJ mol^−1^, and πC3-O5→π*C3-O5 with 0.88 kJ mol^−1^. For the LP→π* transitions, LP (1) N12→π*C11-O134 exhibited a substantial energy value of 1.94 kJ mol^−1^, while LP (1)N4→ π*C3-O55 showed 0.68 kJ mol^−1^. Regarding LP→σ* transitions, LP (2)O5→σ*C2-C31 demonstrated 9.5 kJ mol^−1^, and LP (1)N4→σ*C7-H24 presented 0.53 kJ mol^−1^. These potent intramolecular hyperconjugative interactions between the π electrons of O–C, O–H, N–C, and C–C bonds and the anti-bonding orbitals of C–C, C–H, and N–C bonds within the ring contribute to the stabilization of specific ring segments, as indicated in Table S6. For compound **5c**, intermolecular hyperconjugative interactions occurred between σC11-H35→σ*N4-C6 and σC15-C16→σ*C15-H39, resulting in stabilization energies of 5.72 kjmol^−1^ and 0.51 kjmol^−1^, respectively. This effect was further enhanced by conjugation with anti-bonding orbitals πC21-C22→π*C20-C251 and πC21-C22→π*C23-C24, leading to significant delocalization energies of 8.74 kjmol^−1^ and 0.08 kjmol^−1^, respectively. The highest energy interaction in the molecule involved electron donation from σC23-C24 to the anti-bonding acceptor σ*C22-Cl2, with a stabilization energy of 5.39 kjmol^−1^. Regarding LP→π* transitions, LP (1)N13→π*C10-O144 and LP (1)N4→π*C8-C9 exhibited stabilization energies of 9.53 kjmol^-l^ and1.16 kjmol^−1^ (Table 2). In compound **5d**, the πC10-O14→π*C10-O14 transition displayed the maximum stabilization energy of 1.38 kjmol^−1^, while the πC3-O5→π*C3-O5 transition exhibited the lowest stabilization energy at 0.91 kjmol^−1^. The electronic transition LP (2)O5→ σ*C2-C31 showed a maximum stabilization energy of 8.8 kjmol^−1^, whereas LP (2)C26→ σ*C23-C24 had the lowest stabilization energy, 0.5 kjmol^−1^. The electronic transition of the LP (1)N4→π*C3-O5 observed the highest stabilization energy 58.98 kjmol^−1^ while LP (2)O14→π*C3-O5 exhibited the lowest, at 0.57 kjmol^−1^.

### 2.4. Nonlinear Optical (NLO) Properties

Nonlinear optical materials have been extensively investigated for their significant contribution to modern optoelectronic and optical signal processing, optical imaging, and optical communications. Organic compounds with a variety of substituents have been explored for their NLO potential [46,47,48]. The experimental evaluation of the NLO response of a large number of compounds is quite challenging and expensive. Therefore, a low-cost solution for the evaluation of the NLO response of a large variety of compounds is the use of quantum chemical calculations [46]. The emphasis on organic compounds **5a**–**5d** stemmed from their affordability, adjustable absorption spectra, and significant nonlinear optical response [49,50]. The electronic characteristics, believed to govern the intensity of these optical responses, were influenced by the overall molecular dipole moment, polarizability, and hyperpolarizabilities [51]. These properties were computed, and the resulting data are presented in (Table 3). The NLO properties of the target compounds **5a**–**5d** were calculated using the B3LYP/6-311G(d,p) level. Equations (2)–(4) determine the nonlinear optical parameter [52].

(2) $$\mu_{total}=\left(\mu_{x^{2}}+\mu_{y^{2}}+\mu_{z^{2}}\right)\frac{1}{2}\;$$

(3) $$\alpha_{mean}=\frac{1}{3}(\alpha_{xx}+\alpha_{yy}+\alpha_{zz})$$

(4) $$\beta_{total}=[{\left(\beta_{xxx}+\beta_{yyx}+\beta zzx\right)}^{2}+{\left(\beta_{yyy}+\beta_{xxy}+\beta_{zzy}\right)}^{2}+{\left(\beta_{zzz}+\beta_{yyz}+\beta_{xxz}\right)}^{2}]\frac{1}{2}$$

In the titled compounds **5a**–**5d**, the linear polarizabilities along the x, y, and z axes were obtained as follows: (235.17, 194.83,156.55 a.u), (205.52, 144.17, 145.25 a.u), (287.22, 203.77, 211.25), and (176.52, 168.30, 147.65, a.u), respectively, leading to α _total_ values of 195.52, 164.98, 234.08, and 164.16 a.u., respectively. Hence, the order of linear polarizability observed for the synthesized compounds was **5c** > **5a** > **5b** > **2d**. The results of hyperpolarizability along the main contributing variables for compound **5a** were obtained as follows: βxxx = 3.13, βxxy = −40.18, βxyy = −32.04, βyyy = −193.56, βxxz = −73.64, βxyz = −3.40, βyyz = 46.84, βxzz = 73.13, βyzz = 52.72, βzzz = 62.49, and βtotal = 189.73. For compound **5b**, the results of hyperpolarizability were βxxx = −61.39, βxxy = −46.79, βxyy = −23.34, βyyy = −114.48, βxxz = 113.62, Βxyz = −30.76, βyyz = −60.01, βxzz = 78.86, βyzz = 23.64, βzzz = −83.62, and β_total (a.u)_ = 140.98. For compound **5c**, the results were βxxx = −144.50, βxxy = 15.73, βxyy = 73.94, βyyy = −18.17, βxxz = −66.34, Βxyz = 31.33, βyyz = −75.51, βxzz = 15.06, βyzz = 13.76, βzzz = -204.15, and βtotal = 350.60. For compound **5d**, the results were βxxx = −52.17, βxxy = −44.23, βxyy = −92.15, βyyy = −99.49, βxxz = 98.06, Βxyz = 18.09, βyyz = 14.56, βxzz = 0.77, −βyzz = 39.83, βzzz = −49.65, and βtotal (a.u) = 241.37. Consequently, compound **5c** possesses the highest hyperpolarizability value, followed by compounds in the decreasing order of **5a** > **5b** > **5d**. Compound **5c** possesses a halogenated phenyl group, which is a larger size compared to **5a**, **5b**, and **5c**. Owing to the larger size, the electron cloud of compound **5c** is more delocalized than in the other compounds, which is the primary reason for its higher polarizability and hyper-polarizability [53].

The experimental and theoretical NLO responses of some related compounds have been reported in the literature [46,54]. Table 3 presents a direct comparison between the NLO responses of the synthesized compounds to those reported in the literature. The analysis indicated that compound **5c** is a promising candidate for enhanced NLO response compared to previously reported compounds with similar structural characteristics. The calculated polarizability and hyperpolarizability values for compound **5c** were 34.69 Å, 2.99 × 1^−28^ e.s.u, and 350.60 (a.u), 3.02 × 10^−27^ esu, respectively. These values are notably higher than those of urea, which are 3.884D (dipole moment), 5.041 Å (polarizability), and 0.782 × 10 − 30 e.s.u. (hyperpolarizability). Urea is often used as a benchmark in NLO studies [55]. The significant increase in the polarizability and hyperpolarizability of compound **5c** indicates that it has excellent NLO properties and considerable potential for use in optoelectronic applications.

### 2.5. FMO Analysis

**Figure 5:**
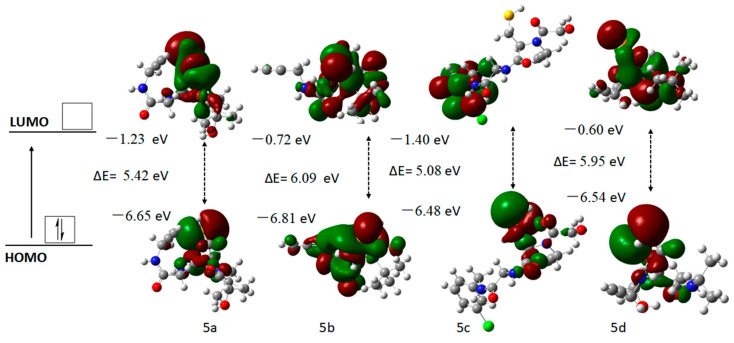
Three-dimensional models of FMOs of the compounds (**5a**–**5d**) computed at B3LYP/6-311G(d,p) level.

FMO (frontier molecular orbital) analysis was used as a significant tool for determining chemical reactivity, optical properties, charge transfer, chromophore interaction, and chemical stability. The HOMO exhibited the capacity to donate electrons while the LUMO demonstrated the ability to accept them [52,56]. The energy band gap was calculated by using Equation (5) [57]. Compounds **5a**–**5d** with larger HOMO–LUMO energy gaps were regarded as more rigid and less reactive, and they exhibited outstanding kinetic stability, while those with smaller gaps were considered softer compounds with enhanced polarizability and greater susceptibility to chemical reactions, particularly aromatic substitution processes. The energies of compounds **5a**–**5d** were computed at the B3LYP/6-311G(d,p) level to assess energy gaps (∆E) between FMOs (Figure 5 and Table 4).

(5) $$Energy\;gap=\left(E_{LUMO}-E_{HOMO}\right)\;eV$$

The computed HOMO–LUMO energy gaps of target compounds **5a**–**5d** were found to be 5.42, 6.09, 5.08, and 5.95 eV, respectively. Among them, compound **5c** exhibited the lowest energy gap of 5.08 eV. This reduced energy gap was likely due to the increased resonance effect caused by the electron-withdrawing nature of the 3-chlorobenzyl group at the acceptor site, which also exhibits an inductive (I) effect. A smaller energy gap typically indicates higher reactivity and lower stability, whereas a larger gap suggests the opposite. Therefore, compound **5c** demonstrated a better ability to easily transfer electron density and behaved as a highly reactive compound. Koopman’s theorem was employed to calculate molecular properties, including electronegativity (X), electron affinity (A), global softness (σ), ionization potential (I), and hardness (η), for compounds **5a**–**5d** based on HOMO and LUMO energies [58,59]. Koopman’s theorem was employed to estimate the ionization potential (IP) and electron affinity (EA), with IP approximated as E_HOMO_ and EA as E_LUMO_. It is important to note that this method offers only a rough estimation, as it overlooks electron correlation and orbital relaxation. Particularly, the energies of virtual orbitals, like ELUMO, are recognized as being less dependable, and the sign of the calculated EA should be interpreted with caution [60,61].

Electron affinity and ionization potential were utilized to characterize the electron-donating and accepting capabilities of the molecules. The title compound **5c** exhibited an ionization potential (I) that was considerably greater than its electron affinity (A) (Table 4). Equations (6) and (7) were employed to determine the electron affinity and ionization potential [59].

(6) $$I=-E_{HOMO}$$

(7) $$A=-E_{LUMO}$$

In this context, Equations (8)–(10) were used to calculate global hardness (g), electronegativity (X), and chemical potential (l) [62].

(8) $$\eta =\frac{I-A}{2}$$

(9) $$X=\frac{I+A}{2}$$

(10) $$\mu =-\frac{I+A}{2}=-X$$

To assess charge transfer in compounds (**5a**–**5d**), the electrophilicity (ω) was determined using Equation (11) [63].

(11) $$\omega =\frac{\mu^{2}}{2\eta }$$

Equation (12) can be utilized to determine the global softness (σ) [64].

(12) $$\sigma =\frac{I}{2\eta }\;$$

### 2.6. PDOS and TDOS Topology

**Figure 6:**
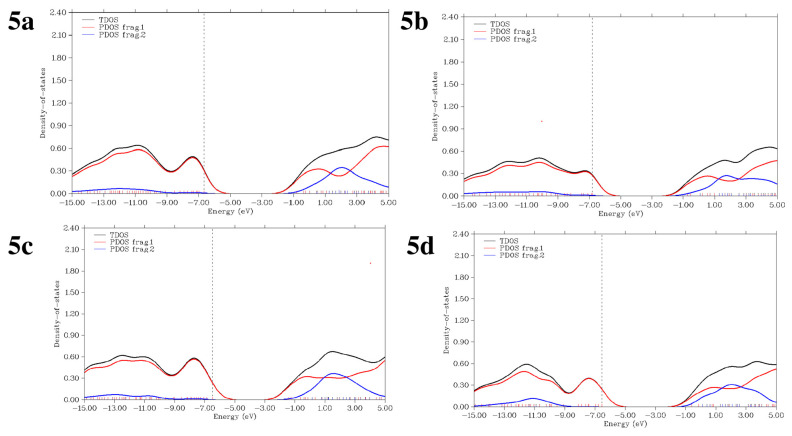
TDOS and PDOS spectra of compounds **5a**–**5d**.

A density of states (DOS) analysis was used to examine the frequency of electronic excitations per unit of energy and volume. To evaluate various electronic activities, such as the total electron scattering, excitation, and λmax of new materials, it is essential to comprehend the probability of potential states per unit volume and energy. The electrical properties between HOMOs and LUMOs vary due to the presence of different electron-activating and deactivating groups in the compounds. Positive x-axis values represent the electrical configuration of LUMOs while negative values indicate the conductive channel at the HOMO. The difference between these values reflects the corresponding energy gap. The calculated total density of electronic states (TDOS) and the partial density of states illustrate the contributions of the two fragment orbitals to the atomic orbital, as shown in Figure 6. In the PDOS graph, 0 eV marks the Fermi level, where the HOMO and LUMO overlap and charge transfer occurs. A versatile wave function analysis program was used to perform several analyses, such as generating TDOS and PDOS fragment graphs [65]. Figure 6 illustrates the computed TDOS and PDOS.

### 2.7. ELF and LOL Methodology Analysis

**Figure 7:**
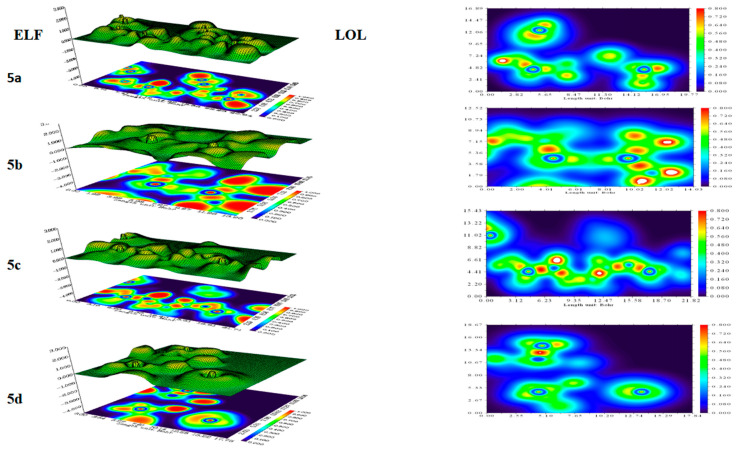
ELF and LOL analysis of title compounds **5a**–**5d**. UV–visible analysis.

The electron localization function and localized orbital locator were utilized to evaluate the distribution of electron density within the compounds. An analysis of the electron localization function (ELF) and localized orbital locator (LOL) was employed to evaluate the distribution of the electron density within the desired compounds (**5a**–**5d**) [66]. These methods were applied to both surface and covalent bond analyses using ELF, employing a bump map and LOL map to examine the atomic region. A bump map, featuring a broad or narrow peak, reflected the electronic environment around an atom. These analyses provided a valuable chance to pinpoint electron pairs on the molecular surface [67]. Figure 7 displays the ELF and LOL of the title compounds **5a**–**5d**. The images revealed that covalent regions exhibited high LOL values, shown in red, while blue circles around the nuclei highlighted areas of electron depletion between valence and inner shells. Core regions were characterized by circular localization zones with elevated electron localization values, which were also shown in red. The carbon–nitrogen (C–N) and carbon–oxygen (C–O) chemical bonds are depicted by irregular localization regions (orange) with electron localization results ranging from 0.16 to 0.5. Both bonds demonstrated covalent characteristics and significant electron localization, which are characteristic of covalent bonding. The ELF map showed that the regions surrounding carbon and nitrogen exhibited lower values, which implies electron delocalization. In contrast, the regions near hydrogen atoms displayed relatively higher values, indicating the presence of both bonding and non-bonding localized electrons. In the LOL figure, the red color was distinctly visible, spreading into the interstitial space among the boundary atoms. The NBO analysis indicated that the stabilization energy for C–N bonds was low, which was also depicted in the LOL figure by relatively low values, as seen in the distortion between nitrogen–carbon atoms.

### 2.8. UV–Visible Spectroscopy

**Figure 8:**
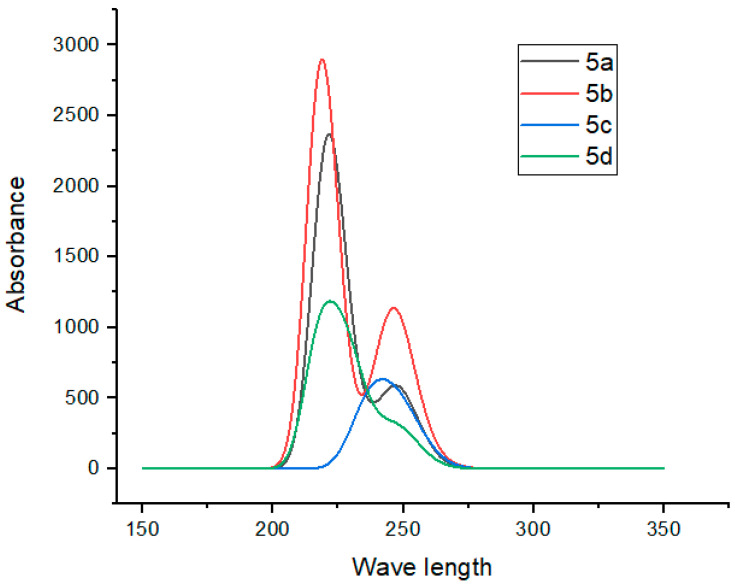
Graphic illustrating the UV–Vis spectroscopy analysis of the target compounds **5a**–**5d**, representing the arbitrary units (molar absorbance coefficient M^−1^cm^−1^).

The theoretical UV–Vis spectrum of compounds **5a**–**5d** was calculated in the chloroform solution. These absorptions were computed with the TD-DFT B3LYP/6-311G(d,p) level of theory [59,68]. The absorption intensities are presented in arbitrary units (molar absorbance coefficient M^−1^cm^−1^ as derived from oscillator strengths). The theoretically (TD-DFT) calculated excitation energies, absorption wavelengths, and oscillator strengths in the absorption spectrum of the title compounds **5a**–**5d** are depicted in Table 5. The molecular orbitals involved in the absorption spectrum of compounds **5a**–**5d** are displayed in Figure 8. The UV–visible absorption range for target compounds **5a**–**5d** was observed between 238 and 296 nm, with peak broadening around 245 nm attributed to the solvent effect (chloroform). In compound **5a**, the maximum absorption wavelength was found to be 255 nm based on DFT calculations, which closely correlates with the experimental value of 278 nm. This absorption was attributed to the C=O group. The oscillator strength was low (f = 0.0003), with the major contributions to the transition being 45% from the H2→LUMO and 37% from the H1→LUMO. For compound **5b**, the calculated maximum wavelength was 257 nm, which aligns well with the experimental value of 296 nm. This absorption was attributed to the C=O group. The oscillator strength was slightly higher (f = 0.0006), with a major contribution of 63% from the HUMO→LUMO+1 (π→*π) transition and a minor 12% contribution from the same transition, indicating relatively stronger allowed transitions. For compound **5c**, the calculated absorption wavelength was 232 nm, closely corresponding to the experimental value of 238 nm. This absorption was attributed to the Cl group. The oscillator strength was low (f = 0.0002), with a major contribution of 86% from HOMO→LUMO indicating weakly allowed n→*σ transitions. In compound **5d**, the absorption wavelength was calculated to be 243 nm, aligning well with the experimental value of 248 nm. This absorption was attributed to the C=O group. The oscillator strength was (f = 0.0022) and the π→*π transition was mainly 81% from HOMO→LUMO, suggesting a weakly allowed but more significant transition than in the other compounds. It is important to mention that the simulated spectra in Figure 8 show a dominant absorption at around the 200 nm plus region (possibly due to S_0_→S_2_), indicating either elevated oscillator strength for S_2_ or limitations in the theoretical method (e.g., missing solvent effects). Additionally, the bandwidths were generated using GaussView’s default spectral simulation function. Additionally, the S_0_→S_1_ transition’s near-zero oscillator strength is responsible for negligible absorbance, indicating the challenges of experimentally detecting symmetry-forbidden transitions. This limitation probably arises due to the simulated spectra representation in relative state-to-state intensities instead of the absolute absorbances. Furthermore, such transitions are not common to observe and may only become observable under specific conditions, such as through vibronic coupling, solvent perturbations, or low-temperature spectroscopy.

### 2.9. Natural Population Results

**Figure 9:**
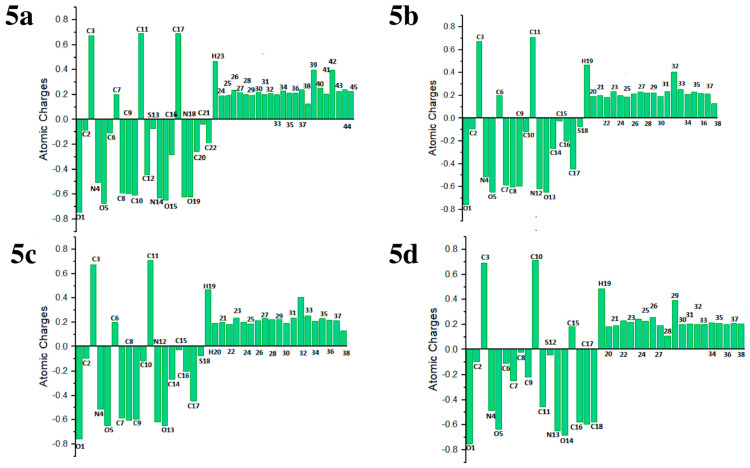
Natural population analysis of the compounds **5a**–**5d**.

NPA analysis was carried out through the B3LYP/6-311G(d,p) basis set of theory to explore the charge distribution in the title compounds **5a**–**5d**. NPA was conducted to ascertain the charge on each atom within the specified compounds. The NPA calculations were performed using Gaussian 09 software. For compound **5a**, the most significant negative charge (−0.75867e) was located on the oxygen atom O1. In compound **5b**, the highest negative charge (−0.67547e) was observed on the oxygen atom O5. Similarly, in compound **5c**, the largest negative charge (−0.64986e) was found on the oxygen atom O13. Compound **5d** also showed its most negative charge (−0.64855e) on the oxygen atom O15. However, across all four compounds, the maximum positive charge was consistently found on the carbon atom C10 (0.71241e), attributed to its direct bonding with two highly electronegative nitrogen atoms. Specifically, the positive charge values were 0.67408e on C3 in compound **5a**, 0.71241e on C10 in **5b**, 0.70652e on C11 e in **5c**, and 0.68913e on C17 in **5d**, as shown in Figure 9.

### 2.10. MEP Surface Analysis

**Figure 10:**
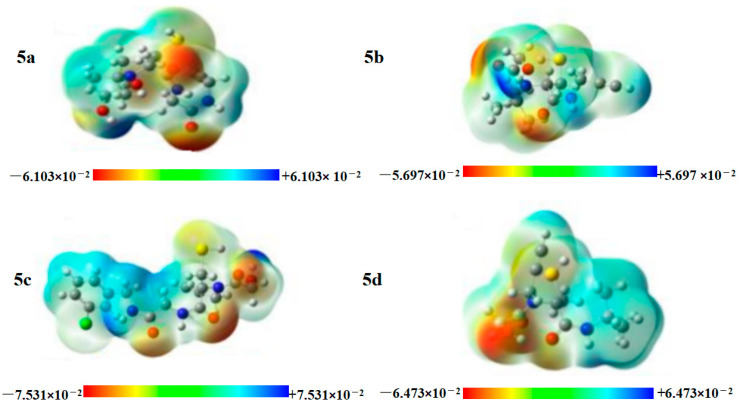
MEP analysis of the title compounds **5a**–**5d** computed at B3LYP/6-311G(d,p). The color coding is shown in the scales. The values in the scales are in Hartree.

MEP analysis proved to be a valuable tool for visualizing molecular size, shape, and charge distribution. In recent years, MEP maps were extensively used to examine H-bond interactions and biological recognition processes and to identify reactive sites for nucleophilic and electrophilic attacks during chemical reactions [69]. MEP was associated with the distribution of a compound’s total charge and can provide insights into various molecular physical properties, including electronegativity, chemical reactivity, dipole moment, and atomic partial charges. In the MEP diagram, different colors (blue, green, red, and yellow) correspond to varying electrostatic potential values on the surface, from electron-rich to electron-poor regions. Blue indicates regions with the highest positive electrostatic potential, red shows regions with the highest negative potential, green represents areas of zero potential, and yellow zones are neutral zones. These color variations reflect different electron densities and charge distributions, with red indicating electron-rich sites and blue representing the electron-poor areas of molecules. Figure 10 displays the MEP plots for compounds **5a**–**5d**. These plots were particularly useful for the three-dimensional visualization and understanding of molecular geometry. Throughout the entire molecule, the areas with electron densities surpassing that of the nucleus are the most negatively charged, and the image reveals that these areas are predominantly located around the O atoms. Positive potentials are primarily found near H atoms. The MEP image (B3LYP/6-311+G(d,p)) was derived from the optimized geometry. Nonetheless, regions of positive electrostatic potential are also present to some extent on CH atoms.

## 3. Experimental

### 3.1. Chemistry

The chemicals required for the synthesis of these desired peptoids were purchased from Acros Chemicals (Geel, Belgium), BDH (Mumbai, India), Fisher Scientific Ltd. (Waltham, MA, USA), Sigma-Aldrich (St. Louis, MO, USA), etc., and were used as received. To check the progress of the reaction, TLC (thin layer chromatography) was conducted using pre-coated silica gel plates and visualized under ultraviolet light at 254 nm and by staining with vanillin in sulfuric acid. For the structural analysis of the peptoids, nuclear magnetic resonance (NMR) spectroscopy was employed, using the Bruker-Avance A-Vspectrometer (operating at 400 MHz for ^1^H and 100 MHz for ^13^C NMR, Bruker, Billerica, MA, USA) to capture NMR spectra in deuterated solvents.

### 3.2. General Method for the Synthesis of Peptoids

The general Ugi reaction procedure involved the addition of carbonyl and amine components first in a round bottom flask having methanol as solvent. Next, carboxylic acid was added in order to activate the imine to imnium ions; then, the isocyanide component was added in a one-pot manner to generate the desired peptoids [1,10]. Accordingly, a mixture of 1,4-Dithiane-2,5-diol 1, acting as the carbonyl components (0.5 mmol, 0.5 equiv.), amine 2 (1.2 mmol, 1.2 equiv.), isocyanide 3 (1.0 mmol, 1.0 eq.), and 2-hydroxyacetic acid or glycolic acid 4 (1.2 mmol, 1.2 eq.) were added to a round bottom flask and stirred in methanol (10 mL) at r.t. for 12 h. Upon completion of the reaction (monitored by TLC), the crude product was purified by using column chromatography in order to obtain the target peptoid using n-hexane–ethylacetate solvent system.

### 3.3. Synthesis of 2-(N-(Tert-butyl)-2-hydroxyacetamido)-3-mercapto-N-(2-oxo-2-(prop-2-yn-1-ylamino) Ethyl) Propenamide (***5a***)

**Scheme 1:**
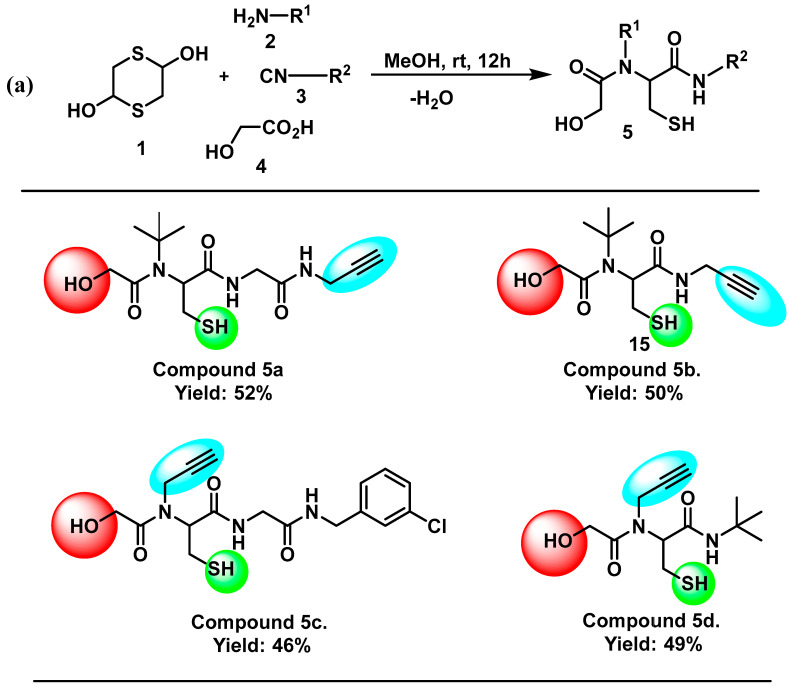

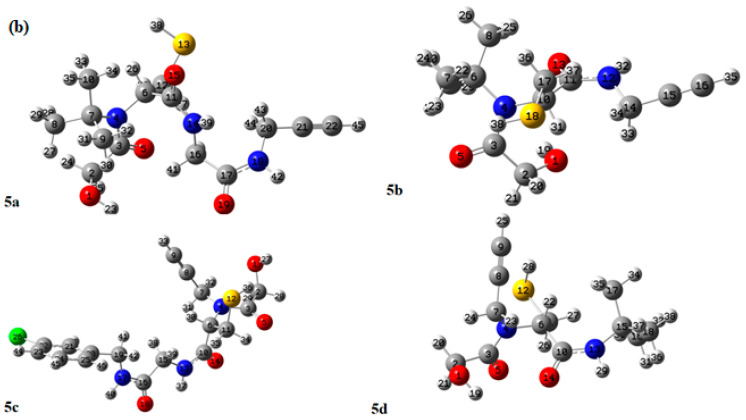
(**a**) Synthesis of new 1,4-dithiane-2,5-diol-based peptoids using Ugi 4-CR approach. (**b**) Optimized structures of compounds **5a**, **5b**, **5c**, and **5d** using Gaussian software.

Compound **5a** was synthesized following the general procedure using 1,4-Dithiane-2,5-diol **1** (0.5 mmol, 0.5 equiv.), tert-butylamine **2** (1.2 mmol, 1.2 equiv.), 2-isocyano-*N*-(prop-2-yn-1-yl) acetamide **3** (1.0 mmol, 1.0 eq.), and 2-hydroxyacetic acid **4** (1.2 mmol, 1.2 eq.), which were stirred in methanol (10 mL) at r.t. for 12 h. Upon completion of the reaction, the final product **5a** was isolated in 52% yield by using column chromatography (Scheme 1).

^1^H NMR (400 MHz, CDCl_3_) δ 8.25–7.89 (m, 1H), 6.74–6.55 (m, 1H), 4.25–4.14 (m, 2H), 4.10–4.04 (m, 3H), 4.03–3.96 (m, 2H), 3.37 (t, *J* = 5.9 Hz, 2H), 2.25 (dt, *J* = 8.0, 2.5 Hz, 1H), 1.32–1.28 (m, 1H), 1.27–1.15 (m, 9H). ^13^C NMR (100 MHz, CDCl_3_) δ 190.0, 171.9, 165.8, 69.3, 65.83, 54.39, 40.30, 36.45, 33.19, 30.86, 26.68, 25.15. EI-MS *m*/*z* 329.0 [M]^+^; IR ύ_max_ 3345, 3083, 2920, 1695, 1595 cm^−1^.

### 3.4. Synthesis of 2-(N-(Tert-butyl)-2-hydroxyacetamido)-3-mercapto-N-(prop-2-yn-1-yl) Propenamide (***5b***)

Compound **5b** was synthesized following the general procedure using 1,4-Dithiane-2,5-diol **1** (0.5 mmol, 0.5 equiv.), tert-butylamine **2** (1.2 mmol, 1.2 equiv.), 3-isocyanoprop-1-yne **3** (1.0 mmol, 1.0 eq.), and 2-hydroxyacetic acid **4** (1.2 mmol, 1.2 eq.), which were stirred in methanol (10 mL) at r.t. for 12 h. Following the completion of reaction, the final product **5b** was isolated in 50% yield using column chromatography (Scheme 1).

^1^H NMR (400 MHz, CDCl_3_) δ 8.01 (s, 1H), 6.06 (s, 1H), 4.56–4.45 (m, 1H), 4.37 (dd, *J* = 4.6, 3.5 Hz, 1H), 3.99 (dd, *J* = 17.3, 2.3 Hz, 1H), 3.47 (d, *J* = 17.0 Hz, 1H), 3.37–3.22 (m, 2H), 3.07 (dd, *J* = 13.7, 4.8 Hz, 1H), 2.33 (t, *J* = 2.4 Hz, 1H), 1.77 (s, 1H), 1.39 (s, 9H). ^13^C NMR (100 MHz, CDCl_3_) δ 167.8, 166.2, 77.9, 73.3, 63.8, 37.4, 31.1, 29.3, 28.9, 28.6. ESI-MS *m*/*z* 295.0 [M + Na]^+^; IR ύ_max_ 3287, 3095, 1735, 1696, 1600 cm^−1^.

### 3.5. Synthesis of N-(2-((3-Chlorobenzyl) Amino)-2-oxoethyl)-2-(2-hydroxy-N-(prop-2-yn-1-yl) Acetamido)-3-mercaptopropanamide (***5c***)

Compound **5c** was synthesized following the general procedure using 1,4-Dithiane-2,5-diol **1** (0.5 mmol, 0.5 equiv.), propargylamine **2** (1.2 mmol, 1.2 equiv.), 3 *N*-(3-chlorobenzyl)-2-isocyanoacetamide **3** (1.0 mmol, 1.0 eq.), and 2-hydroxyacetic acid **4** (1.2 mmol, 1.2 eq.), which were stirred in methanol (10 mL) at r.t. for 12 h. Following thecompletion of reaction, the final product **5c** was isolated in 46% yield using column chromatography (Scheme 1).

^1^H NMR (400 MHz, CDCl_3_) δ 8.0 (d, *J* = 4.9 Hz, 1H), 7.4 (dd, *J* = 18.1, 10.1 Hz, 4H), 6.6 (s, 1H), 4.5 (d, *J* = 5.6 Hz, 1H), 4.4 (t, *J* = 6.9 Hz, 1H), 3.9–3.8 (m, 5H), 3.8–3.7 (m, 1H), 3.4 (dd, *J* = 11.6, 5.0 Hz, 1H), 3.2 (dd, *J* = 11.6, 7.9 Hz, 1H), 2.7–2.6 (m, 2H), 1.7 (t, *J* = 7.9 Hz, 1H), 1.3–1.24 (m, 1H). ^13^C NMR (100 MHz, CDCl_3_) δ 169.7, 165.6, 136.7, 135.1, 132.1, 127.6, 127.0, 126.2, 125.8, 125.3, 125.1, 69.3, 58.5, 41.1, 40.5, 30.8, 29.4. ESI-MS *m*/*z* 397.0 [M]^+^; IR ύ_max_ 3524, 3058, 2924, 1630, 1522.

### 3.6. Synthesis of N-(Tert-butyl)-2-(2-hydroxy-N-(prop-2-yn-1-yl) Acetamido)-3-mercaptopropanamide (***5d***)

Compound **5d** was synthesized following the general procedure using 1,4-Dithiane-2,5-diol **1** (0.5 mmol, 0.5 equiv.), propargylamine **2** (1.2 mmol, 1.2 equiv.), tert-butyl isocyanide **3** (1.0 mmol, 1.0 eq.), and 2-hydroxyacetic acid **4** (1.2 mmol, 1.2 eq.), which were stirred in methanol (10 mL) at r.t. for 12 h. Following the completion of reaction, the final product **5d** was isolated in 49% yield using column chromatography (Scheme 1).

^1^H NMR (400 MHz, CDCl_3_) δ 8.01 (s, 1H), 6.06 (s, 1H), 4.55–4.45 (m, 1H), 4.40 (dd, *J* = 4.6, 3.5 Hz, 1H), 4.00 (dd, *J* = 17.3, 2.3 Hz, 1H), 3.51 (d, *J* = 17.0 Hz, 1H), 3.37–3.22 (m, 2H), 3.07 (dd, *J* = 13.7, 4.8 Hz, 1H), 2.33 (t, *J* = 2.4 Hz, 1H), 1.80 (s, 1H), 1.40 (s, 9H). ^13^C NMR (100 MHz, CDCl_3_) δ 167.9, 166.3, 78.0, 73.3, 63.8, 37.3, 31.2, 29.4, 28.9, 28.5. ESI-MS *m*/*z* 295.0 [M + Na]^+^; IR ύ_max_ 3274, 3062, 2853, 1718, 1653 cm^−1^.

### 3.7. Experimental Section (Characterization, Computational Procedures, DFT-Based Calculations)

The final structures of the desired peptoids (**5a**–**5d**) were elucidated using various spectroscopic techniques. Spectroscopic techniques such as FTIR, UV–Vis, MS, and NMR were used to characterize the targeted peptoids **5a**–**5d**. In the FTIR spectrum of compound **5a**, the appearance of a peak at 3345 cm^−1^ confirmed the presence of an amidic N–H group. A strong absorption peak at 1695 cm^−1^ indicated the presence of the amidic carbonyl functional group in **5a** (Figure S13A). The same pattern was found in the spectra of the other three peptoids as well. The ^1^H NMR spectra of the investigated compounds **5a**, **5b**, and **5d** (Figures S1, S3 and S7) showed the presence of a *tert*-butyl group that had the integration of nine protons. The ^1^H NMR spectrum of compound **5c** showed peaks at around 7.4 to 6.6 ppm, indicating the presence of aromatic protons (Figure S5). The presence of three signals at 190.0, 171.9, and 165.8 ppm in the ^13^C NMR spectrum of compound **5a** (Figure S2), as well as signals at 169.7, 165.6, and 136.7 ppm in **5c** (Figure S6), indicates the presence of three amidic carbonyl carbons. Similarly, two signals at 167.8 and 166.2 ppm in **5b** (Figure S4) and at 167.9 and 166.3 ppm in **5d** (Figure 8) confirm the presence of two amidic carbonyl carbons in these compounds [1,10].

DFT-based calculations were formed using the Gaussian 09 software program [24,70,71] with the B3LYP6-311G(d,p) functional to analyze the quantum chemical properties of compounds **5a**–**5d**. Avogadro 1.2 [72] and GaussView 5.0 [73] were used to prepare the input files. The output files were examined using ChemCraft 1.8 [74] and Gaussian 09W [75]. Additionally, to investigate the optoelectronic properties and the structure–property relationship of the title molecules, NBO, MEP, and FMO analyses were conducted using the B3LYP functional with the 6-311G(d,p) basis set. NBO analysis was conducted using the Gaussian 09W program [76] to identify the hyperconjugations and intermolecular delocalization and to investigate the second-order perturbation interactions between the empty and filled orbitals within the system. FMO analysis was also conducted to assess the energy difference between the orbitals and was further used to evaluate the global reactivity, including the chemical potential (μ), softness (σ), electronegativity (X), and hardness (η). NLO analysis [31,77] of compounds **5a**–**5d** was carried out for the first-order hyperpolarizabilities, dipole moment, and linear polarizability.

## 4. Conclusions

The title compounds **5a**–**5d** were successfully synthesized using a one-pot multicomponent Ugi 4-CR approach with isolated yields of 52%, 50%, 46%, and 49%, respectively. The newly synthesized peptoids were examined through spectroscopic methods like ^13^C and ^1^H NMR and were explored theoretically. According to the FMO analysis, compound **5c** had the lowest energy gap of 5.08 eV, while compounds **5a**, **5b**, and **5d** exhibited higher energy gaps of 5.42, 6.09, and 5.95 eV, respectively. This reduced energy gap in compound **5c** was likely due to the increased resonance effect caused by the electron-withdrawing nature of the 3-chlorobenzyl group at the acceptor site, which also exhibits a negative inductive (-I) effect. A smaller energy gap typically indicates higher reactivity and lower stability. NBO analysis confirmed significant donor–acceptor interactions in compound **5c** (8.74–0.08 kJ/mol), attributed to π→π* transitions, which enhance its intramolecular charge transfer properties. The NPA charge distribution showed that compound **5c** maintains a balanced electron density, contributing to its increased stability. ELF and LOL plots further supported the presence of localized electron pairs and strong covalent bonding in compound **5c**. The highest average polarizability and hyperpolarizability values (determined via the B3LYP/6-311G(d,p) basis set) accompanied their low energy gaps, facilitating strong charge transfer and enhanced NLO properties. The dipole moment of compound **5c** was calculated as 1.12 and 1.23 while the total dipole moment was observed to be 2.59 (a.u). The polarizability values were calculated as 287.22, 203.77, and 211.25, with a total polarizability of 234.08. The hyperpolarizability of the title compound **5c** was found to be −144.50, 15.73, 73.94, −18.17, −66.34, 31.33, −75.51, 15.06, and 13.76, while the total hyperpolarizability was found to be −204.15. The computed polarizability and hyperpolarizability values of compound **5c** were found to be 34.69 Å, 2.99 × 10^−28^ cm^5^/e.s.u and 350.60 (a.u) and 3.02 × 10^−27^ cm^5^/e.s.u Compound **5c** exhibited a marked increase in polarizability and hyperpolarizability, suggesting excellent NLO properties and that it holds significant potential for optoelectronic applications. The newly synthesized peptoids (**5a**–**5d**) bear reactive -OH, -SH, and terminal alkyne groups, providing handles for further structural elaboration into complex derivatives.

## Figures and Tables

**Table 1:** Calculated bond length and bond angle of the target compounds **5a**–**5d**.

Compound	Atom	Bond Length	Atom	Bond Angle
**5a**	O1-H 23	0.97322	O1-C2-C3	114.84476
	C2-C3	1.53016	O5-C3-N4	121.28740
	N4-C6	1.51176	N4-C6-C12	116.23986
	C3-O5	1.26424	C11-C6-C12	115.16739
	N4-C7	1.55457	N4-C7-C9	108.79735
	C7-C8	1.54649	C8-C7-C10	106.27711
	C7-C9	1.54430	C12-C6-C11	115.16739
	C7-C10	1.54908	C6-C12-S13	111.31957
	C6-C11	1.54840	O15-C11-N14	118.96980
	C6-C12	1.54088	N14-C16-C17	111.97091
	C12-S13	1.91628	O19-C17-N18	120.84854
	S13-H38	1.38443	C16-C17-O19	119.98662
	C11-N14	1.36757	N18-C20-C21	113.68073
	N14-C16	1.46795	C20-C21-C22	79.65692
	C16-C17	1.52419		
	C17-N18	1.37023		
	C17-O19	1.25060		
	C20-N18	1.47247		
	C20-C21	1.46846		
	C≡C	1.20534		
**5b**	C2-O1	1.46497	O1-C2-C3	113.81273
	C2-C3	1.53354	C2-C3=O5	115.73547
	C3=O5	1.25559	C3-N4-C6	116.86348
	C3-N4	1.38583	C9-C6-C7	111.08996
	N4-C6	1.55094	C7-C6-C8	107.07582
	C6-C7	1.54816	N4-C10-C11	113.67713
	C6-C8	1.54261	C17-C10-C11	110.50794
	C6-C9	1.54620	N12-C11-O13	120.03093
	N4-C10	1.48668	C10-C11-O13	123.41579
	C10-C17	1.54189	N12-C14-C15	110.39825
	C17-S18	1.90430	C14-C15≡C16	179.54141
	C10-C11	1.54391		
	C11=O13	1.25166		
	C11-N12	1.36754		
	N12-C14	1.48111		
	C14-C15	1.46107		
	C15≡C16	1.20451		
**5c**	O1-C2	1.44950	O1-C2-C3	114.62658
	C2-C3	1.52048	N4-C3=O5	123.33369
	C3-N4	1.36474	N4-C7-C8	112.71823
	C3=O5	1.25019	C7-C8≡C9	178.04819
	N4-C7	1.48093	C6-C11-S12	113.90251
	C7-C8	1.46278	C6-C10=O14	120.80279
	C8≡C9	1.19999	C10-N13-C15	127.69179
	N4-C6	1.46823	C15-C16=O	26.38630
	C6-C11	1.53056	C16-N17-C19	127.50822
	C11-S12	1.87599	C20-C21-C22 (aromatic)	119.16245
	C6-C10	1.53671	C22-C23-C24	118.39558
	C10=O14	1.24338	C24-C25-C20	120.46519
	C10-N13	1.35697		
	C15-C16	1.52011		
	C16=O18	1.24377		
	C16-N17	1.35700		
	C19-C20	1.51215		
	C20-C21 (aromatic)	1.39666		
	C21-C22 (aromatic)	1.38192		
	C22-C23 (aromatic)	1.38493		
	C23-24 (aromatic)	1.38993		
	C24-C25 (aromatic)	1.39155		
	C22-Cl 26	1.81146		
**5d**	O1-C2	1.45474	O1-C2-C3	110.83283
	C1-C3	1.52295	N4-C3=O5	122.42407
	C3=O5	1.24939	N4-C7-C8	112.32576
	C3-N4	1.38758	C7-C8≡C9	178.01041
	N4-C7	1.48327	N4-C6-C10	107.16812
	N4-C6	1.46905	N13-C10=O14	117.85144
	C7-C8	1.46642	C6-C11-S12	113.71090
	C8≡C9	1.20562	N13-C15-C16	109.69455
	C6-C11	1.53943	C17-C15-C18	108.36323
	C6-C10	1.55903	C16-C15-C18	109.18518
	C10-O14	1.26112		
	C10-N13	1.36658		
	N13-C15	1.50181		
	C15-C16	1.54438		
	C15-C17	1.53888		
	C15-C18	1.54742		

**Table 2:** NBO analysis of compound **5c**.

Compound	Donor	Types	Acceptor	Types	E(2) kjmol^−1^	E(j)E(i)(a.u)	F(i.j)(a.u)
**5c**	C11-H35	Σ	N4-C6	σ*	5.72	0.82	0.061
	C15-C16	Σ	C15-H39	σ*	0.51	1.03	0.053
	C21-C22	Π	C20-C251	π*	8.74	0.3	0.067
	C21-C22	Π	C23-C242	π*	0.08	0.3	0.069
	C20-C25	Π	N17-C19	σ*	4.77	0.6	0.052
	C8-C9	Π	C7-H32	σ*	1.09	0.66	0.024
	C7-H31	Σ	C8-C9	π*	4.72	0.59	0.047
	N13-C15	Σ	C10-O14	π*	0.8	0.79	0.024
	O5	LP(2)	C2-C31	σ*	8.8	0.59	0.095
	O1	LP(2)	C2-C3	σ*	7.96	0.66	0.065
	C26	LP(2)	C23-C24	σ*	0.5	0.87	0.019
	N13	LP(1)	C10-O144	π*	9.53	0.29	0.108
	N17	LP(1)	C16-O186	π*	8.66	0.26	0.119
	N4	LP(1)	C8-C9	π*	1.16	0.32	0.019

**Table 3:** NLO properties used for the calculation of compounds **5a**–**5d**.

Parameters	5a (a.s.u)	5b (a.s.u)	5c (a.s.u)	5d (a.s.u)
µ_x_	−4.92 × 10^−30^	8.03 × 10^−30^	−1.71 × 10^−29^	1.76 × 10^−29^
µ_y_	2.59 × 10^−30^	−4.31 × 10^−31^	9.67 × 10^−30^	4.66 × 10^−30^
µ_z_	−3.36 × 10^−30^	−2.24 × 10^−30^	1.06 × 10^−29^	1.14 × 10^−29^
µ_total_	6.47 × 10^−30^	8.29 × 10^−30^	2.23 × 10^−29^	2.15 × 10^−29^
α_xx_	2.03 × 10^−27^	1.77 × 10^−27^	2.48 × 10^−27^	1.52 × 10^−27^
α_xy_	8.33 × 10^−29^	4.81 × 10^−29^	−2.38 × 10^−28^	1.25 × 10^−29^
α_yy_	1.68 × 10^−27^	1.24 × 10^−27^	1.76 × 10^−27^	1.45 × 10^−27^
α_xz_	−1.15 × 10^−28^	−3.94 × 10^−29^	−1.31 × 10^−28^	4.95 × 10^−29^
α_yz_	5.21 × 10^−29^	6.25 × 10^−29^	−5.24 × 10^−29^	9.09 × 10^−29^
α_zz_	1.35 × 10^−27^	1.25 × 10^−27^	1.82 × 10^−27^	1.27 × 10^−27^
α_mean_	1.68 × 10^−27^	1.42 × 10^−27^	2.02 × 10^−27^	1.41 × 10^−27^
Δα	2.50 × 10^−28^	2.11 × 10^−28^	2.99 × 10^−28^	2.10 × 10^−28^
β_xxx_	2.70 × 10^−29^	−5.30 × 10^−28^	1.24 × 10^−27^	−4.50 × 10^−28^
β_xxy_	−3.47 × 10^−28^	−4.04 × 10^−28^	−1.35 × 10^−28^	−3.82 × 10^−28^
β_xyy_	−2.76 × 10^−28^	−2.01 × 10^−28^	6.38 × 10^−28^	−7.96 × 10^−28^
β_yyy_	−1.67 × 10^−27^	−9.89 × 10^−28^	−1.56 × 10^−28^	−8.59 × 10^−28^
β_xxz_	−6.36 × 10^−28^	9.81 × 10^−28^	−5.73 × 10^−28^	8.47 × 10^−28^
Β_xyz_	−2.93 × 10^−29^	−2.65 × 10^−28^	2.70 × 10^−28^	1.56 × 10^−28^
β_yyz_	4.04 × 10^−28^	−5.18 × 10^−28^	−6.52 × 10^−28^	1.25 × 10^−28^
β_xzz_	6.31 × 10^−28^	6.81 × 10^−28^	1.30 × 10^−28^	6.65 × 10^−30^
β_yzz_	4.55 × 10^−28^	2.04 × 10^−28^	1.18 × 10^−28^	−3.44 × 10^−28^
β_zzz_	5.39 × 10^−28^	7.22 × 10^−28^	−1.76 × 10^−27^	−4.28 × 10^−28^
β_total_	1.63 × 10^−27^	1.21 × 10^−27^	3.02 × 10^−27^	2.08 × 10^−27^

**Table 4:** The energy gap between the HOMO and LUMO for targeted compounds **5a**–**5d** was calculated using the B3LYP/6-311G(d,p) computational methods.

Comp	HOMO (eV)	LUMO (eV)	HOMO-LUMO (eV)	I	A	X	η	µ	Ω	σ
**5a**	−6.649	−1.227	5.42	6.65	1.23	3.94	2.71	−3.94	2.86	0.18
**5b**	−6.809	−0.719	6.09	6.81	0.72	3.76	3.04	−3.76	2.33	0.16
**5c**	−6.484	−1.404	5.08	6.48	1.40	3.94	2.54	−3.94	3.06	0.20
**5d**	−6.544	−0.595	5.95	6.54	0.60	3.57	2.97	−3.57	2.14	0.17

**Table 5:** UV–Vis spectroscopy analysis of the target compounds **5a**–**5d**.

Compound	Energy (cm^−1^)	Wavelength (nm) (Cal)	Wavelength (nm) (Exp)	Oscillator Strength	Major Contributions
**5a**	39,069.76	255	278	0.0003	H2-LUMO (45%), H1-LUMO (37%)
**5b**	38,851.18	257	296	0.0006	H1-L+1 (63%), H-L+1 (12%)
**5c**	43,013.04	232	238	0.0002	HOMO-LUMO (86%)
**5d**	41,004.71	243	248	0.0022	HOMO-LUMO (81%)

## Data Availability

The original contributions presented in this study are included in the article/Supplementary Material. Further inquiries can be directed to the corresponding author.

## Supplementary Materials

The following supporting information can be downloaded at: https://www.mdpi.com/article/10.3390/molecules30112340/s1, Figure S1. ^1^H-NMR Spectra of **5a** in CDCl_3_. Figure S2. ^13^C-NMR Spectra of **5a** in CDCl_3_. Figure S3. ^1^H-NMR Spectra of **5b** in CDCl_3_. Figure S4. ^13^ C-NMR Spectra of **5b** in CDCl_3_. Figure S5. ^1^ H-NMR Spectra of **5c** in CDCl_3_. Figure S6. ^13^ C-NMR Spectra of **5c** in CDCl_3_. Figure S7. ^1^ H-NMR Spectra of **5d** in CDCl_3_. Figure S8. ^13^C-NMR Spectra of **5d** in CDCl_3_. Figure S9. MS^2^—spectrum of the [M + H]^+^ion at *m*/*z* 330.0 (**5a**). Figure S10. MS^2^—spectrum of the [M + Na]^+^ ion at *m*/*z* 295.0 (**5b**). Figure S11. MS^2^—spectrum of the [M + H]^+^ ion at *m*/*z* 397.0 (**5c**). Figure S12. MS^2^—spectrum of the [M + Na]^+^ ion at *m*/*z* 295.0 (**5d**). Figure S13. (A) Experimental FTIR spectrum of the compound **5a**, (B) Computed FTIR spectrum of the compound **5a**. Figure S14. (A) Experimental FTIR spectrum of the compound **5b**, (B) Computed FTIR spectrum of the compound **5b**. Figure S15. (A) Experimental FTIR spectrum of the compound **5c**, (B) Computed FTIR spectrum of the compound **5c**. Figure S16. (A) Experimental FTIR spectrum of the compound **5d**, (B) Computed FTIR spectrum of the compound **5d**. Figure S17. Experimental U.V spectrum of the compound **5a**. Figure S18. Experimental U.V spectrum of the compound **5b**. Figure S19. Experimental U.V spectrum of the compound **5c**. Figure S20. Experimental U.V spectrum of the compound **5d**. Table S1. Calculated FTIR analysis of compound **5a**. Table S2. Calculated FTIR value of compound **5b**. Table S3. Calculated FTIR value of compound **5c**. Table S4. Calculated FTIR value of compound **5d**. Table S5. Natural bond orbital (NBO) analysis of compound **5a**. Table S6. Natural bond orbital (NBO) analysis of compound **5b**. Table S7. Natural bond orbital (NBO) analysis of compound **5c**. Table S8. Natural bond orbital (NBO) analysis of compound **5d**.
