# Supplementary material for: One-Pot Approach Towards Peptoids Synthesis Using 1,4-Dithiane-2,5-Diol via Multicomponent Approach and DFT-Based Computational Analysis
Source: Molecules. 2025 May 27;30(11):2340. doi: 10.3390/molecules30112340 (PMC12156225; doi:10.3390/molecules30112340)
Supplement: Supplementary file 1 [file molecules-30-02340-s001.zip › molecules-3637165-supplementary.pdf]

Supporting Information

One-Pot Approach Towards Peptoids Synthesis Using 1,4-Dithiane-2,5-Diol *via* Multicomponent Approach and DFT-Based Computational Analysis

Musrat Shaheen and Akbar Ali *

Department of Chemistry, Government College University Faisalabad, Faisalabad 38000, Pakistan; musarat7734@gmail.com
* Correspondence: akbarchm@gmail.com or akbarali@gcuf.edu.pk

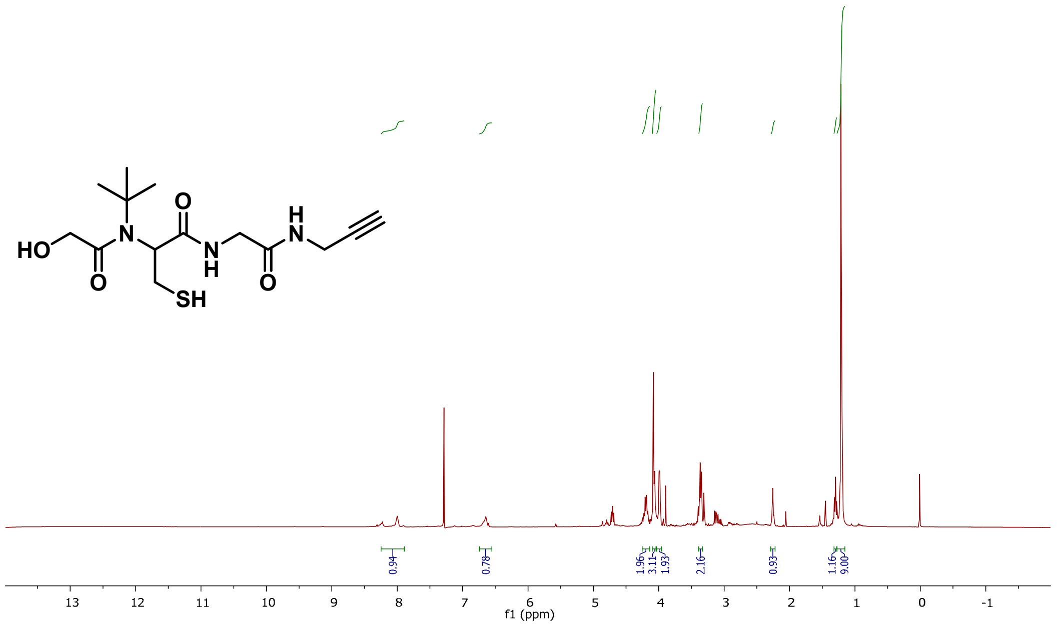

Figure S1 ^{1}H-NMR Spectra of 5a in $CDCl_3$.

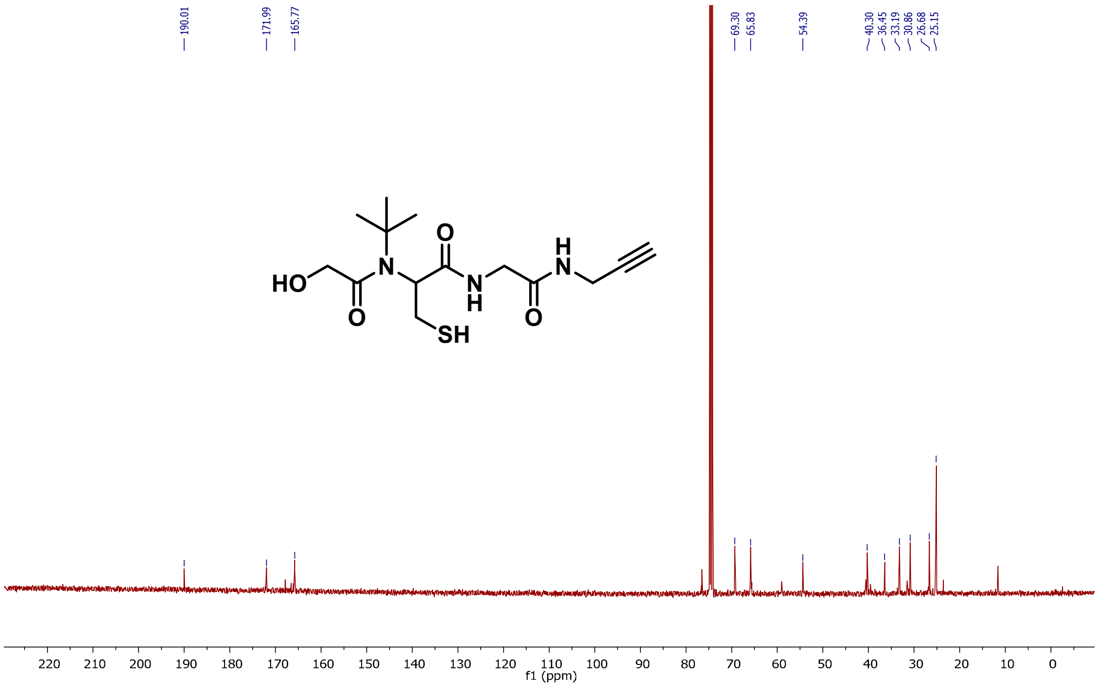

Figure S2 ^{13}C-NMR Spectra of 5a in $CDCl_3$.

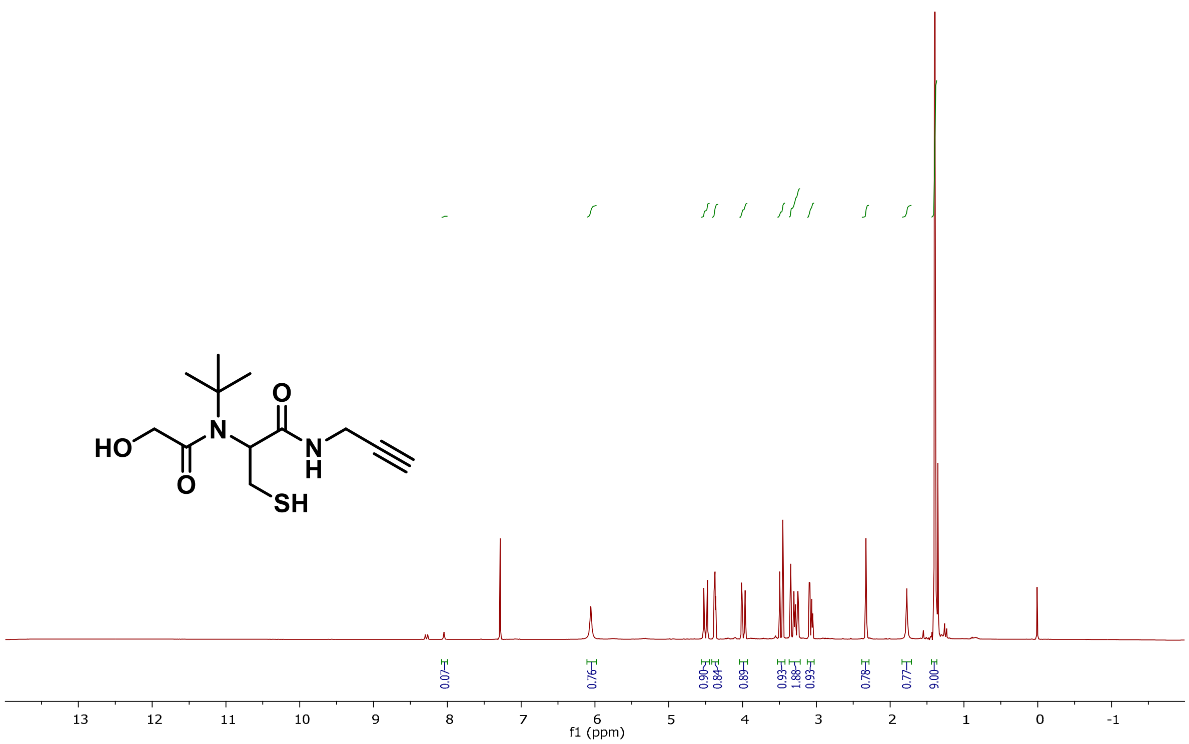

Figure S3 ^{1}H-NMR Spectra of 5b in $CDCl_3$.

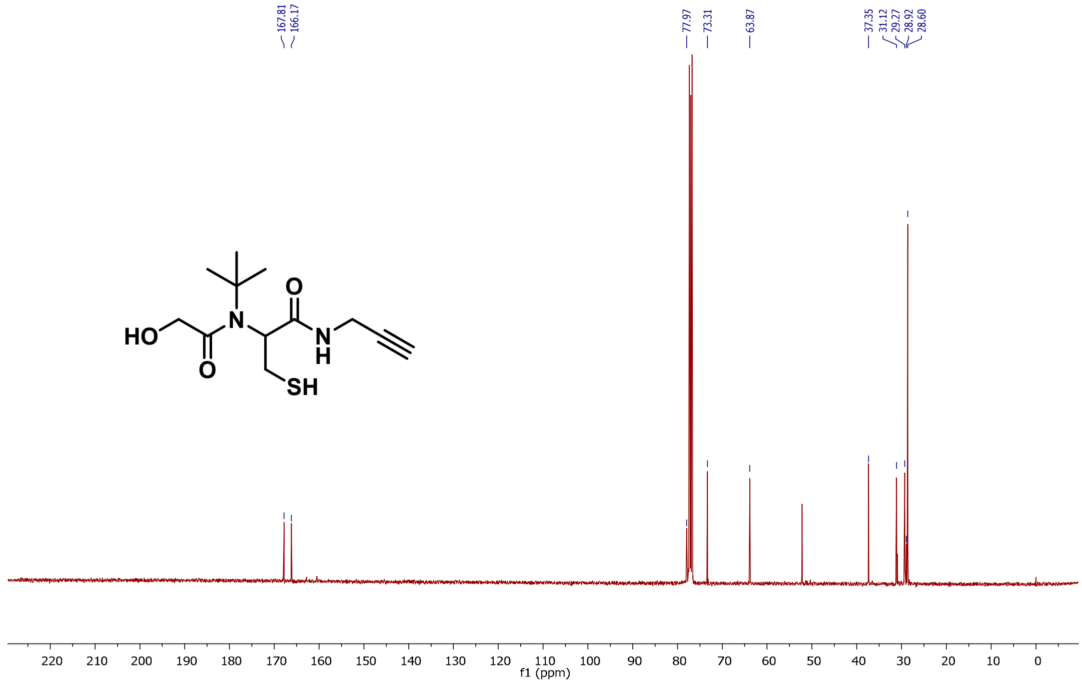

Figure S4 ^{13} C-NMR Spectra of 5b in $CDCl_3$.

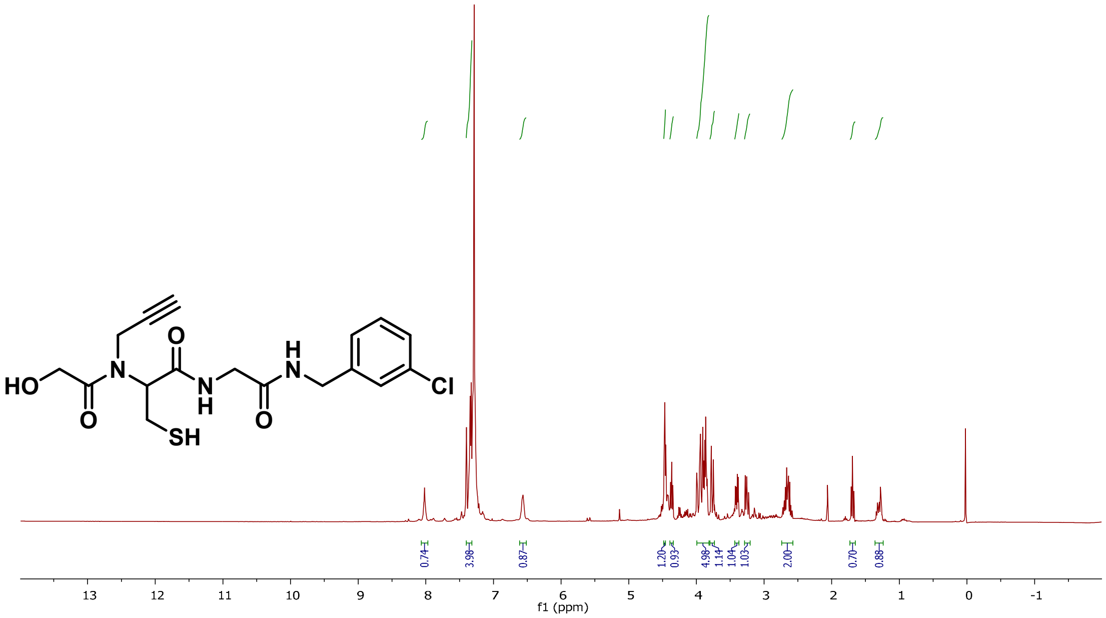

Figure S5 [1] H-NMR Spectra of 5c in $CDCl_3$.

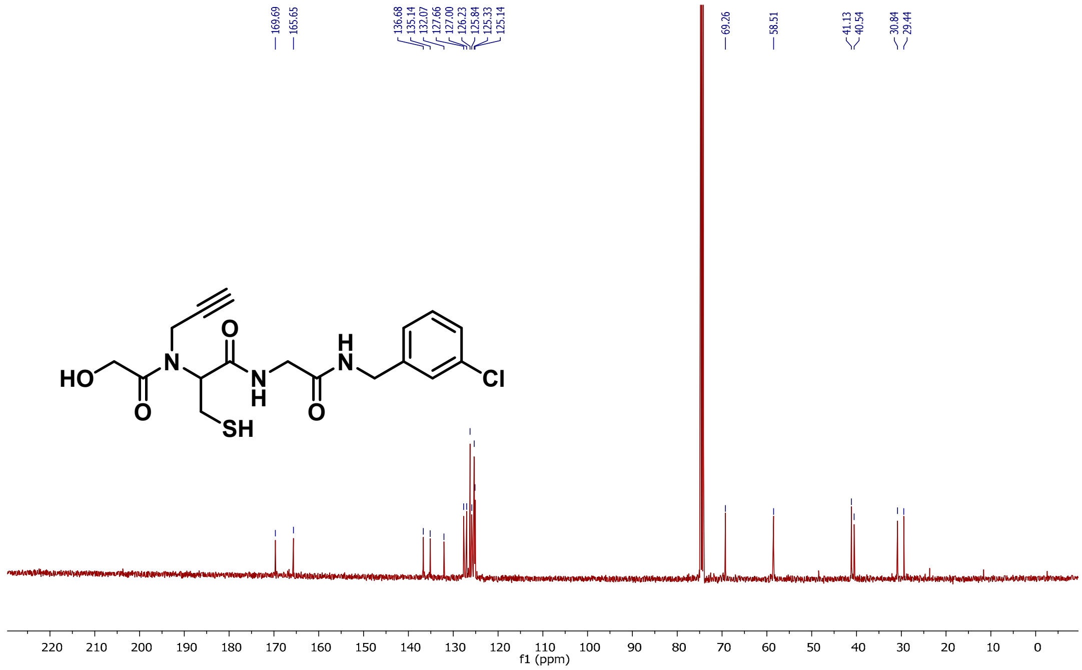

Figure S6 [13] C-NMR Spectra of 5c in $CDCl_3$.

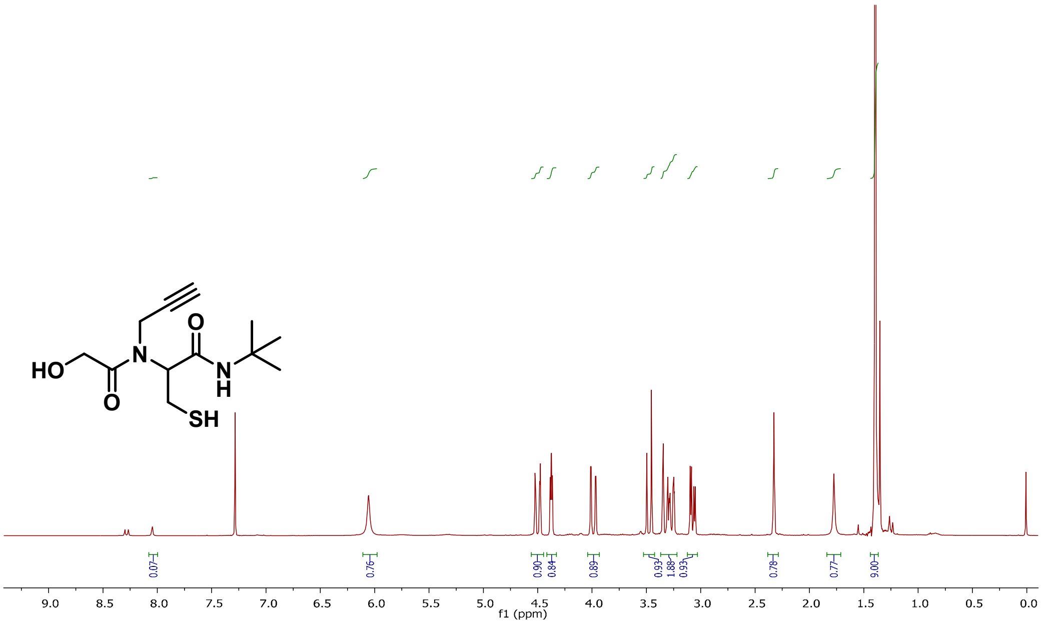

Figure S7 [1] H-NMR Spectra of 5d in $CDCl_3$.

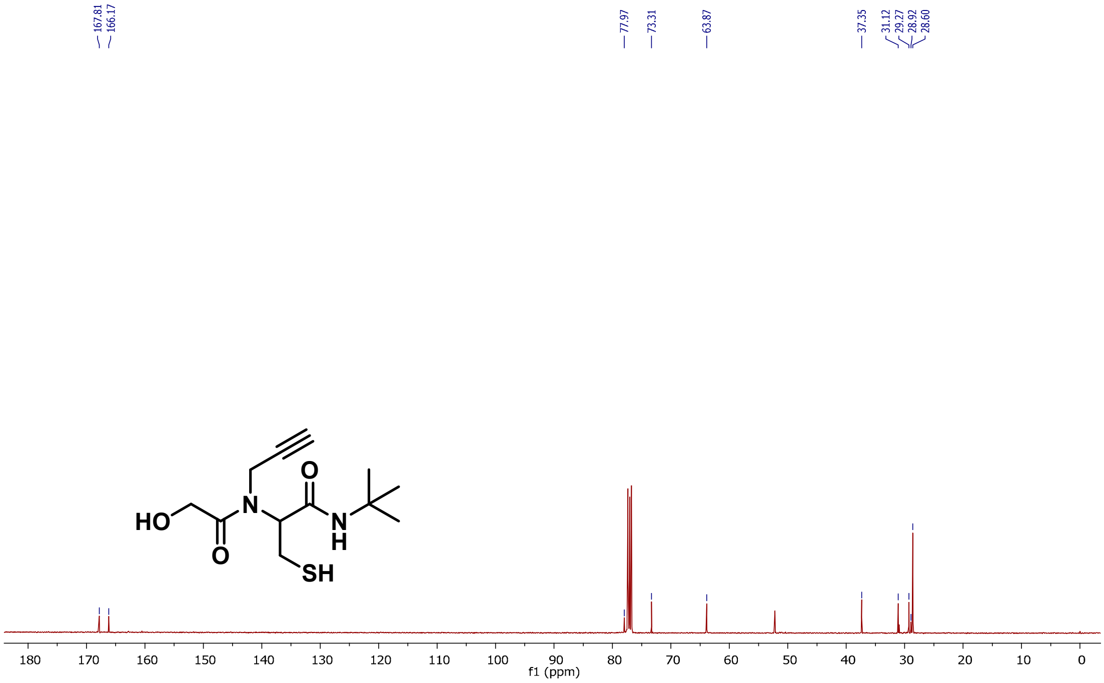

Figure S8 ^{13}C-NMR Spectra of 5d in $CDCl_3$.

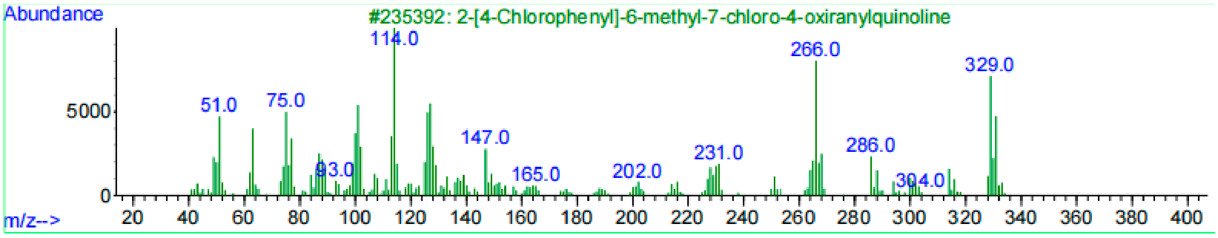

Figure S9 MS^2 - spectrum of the $[M+H]^+$ion at *m/z* 330.0 (5a).

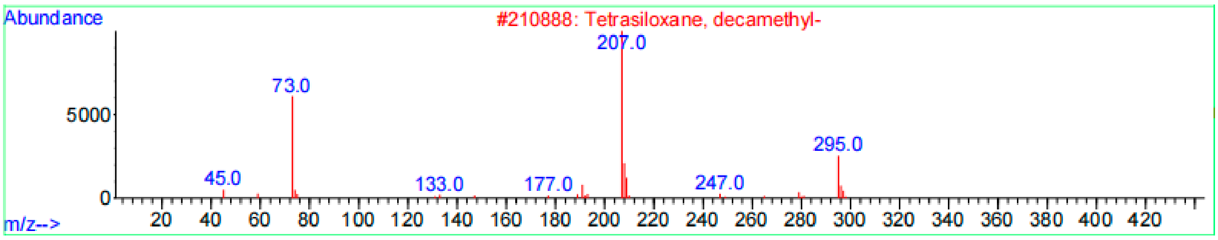

Figure S10 MS^2 - spectrum of the $[M + Na]^+$ ion at *m/z* 295.0 (5b).

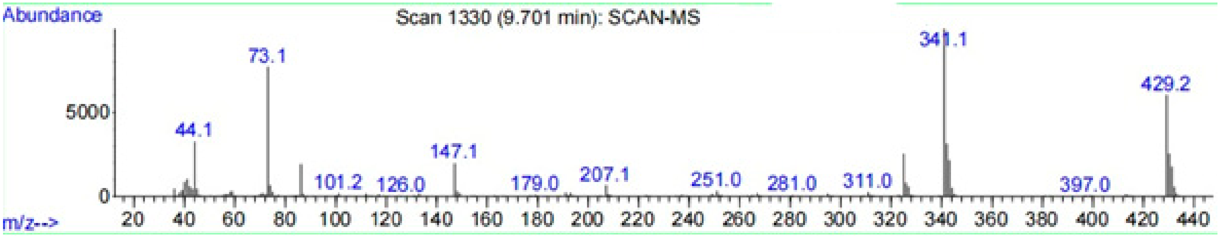

Figure S11 MS^2 - spectrum of the $[M+H]^+$ ion at *m/z* 397.0 (5c).

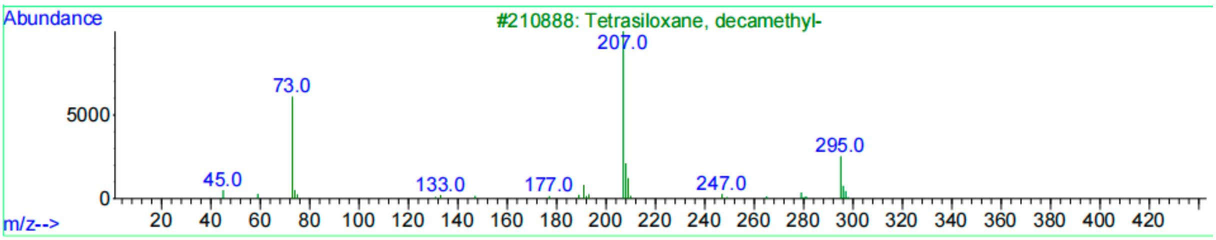

Figure S12 MS^2 - spectrum of the $[M + Na]^+$ ion at *m/z* 295.0 (5d).

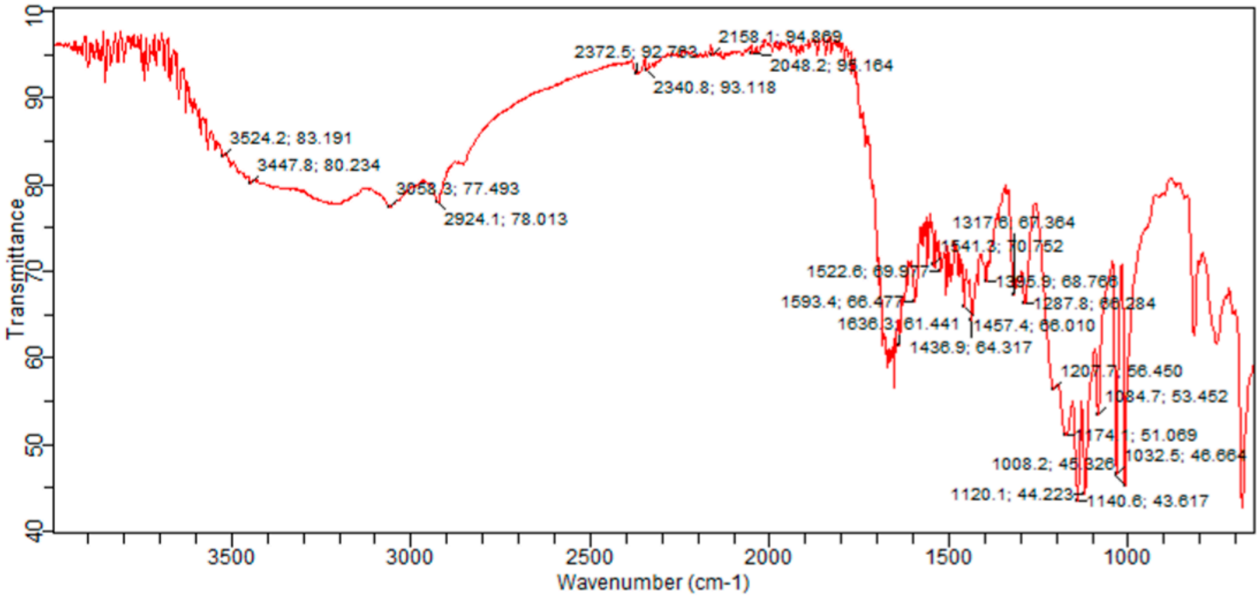

Figure S13-A Experimental FTIR spectrum of the compound 5a

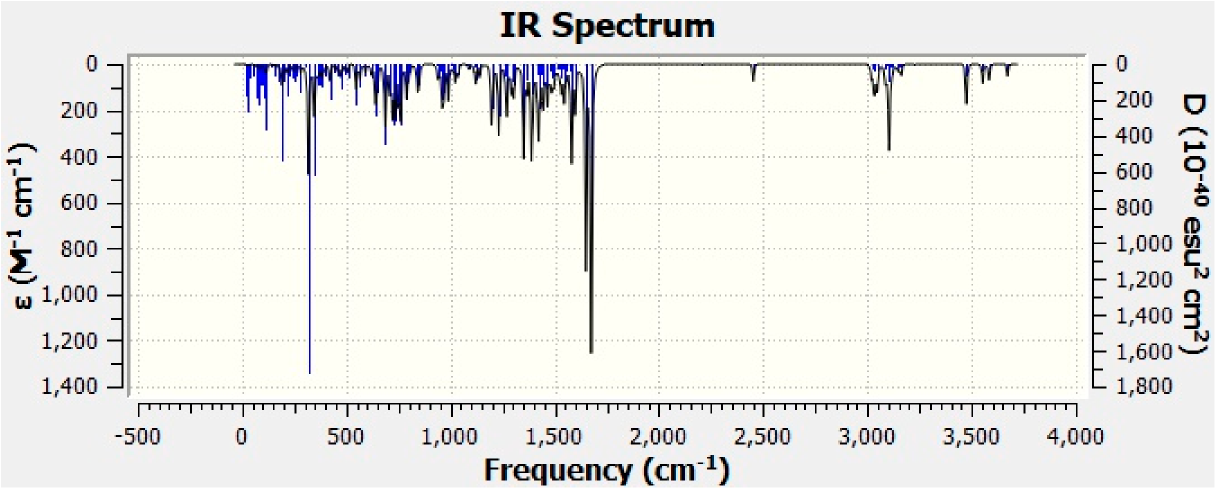

Figure S13-B Computed FTIR spectrum of the compound 5a

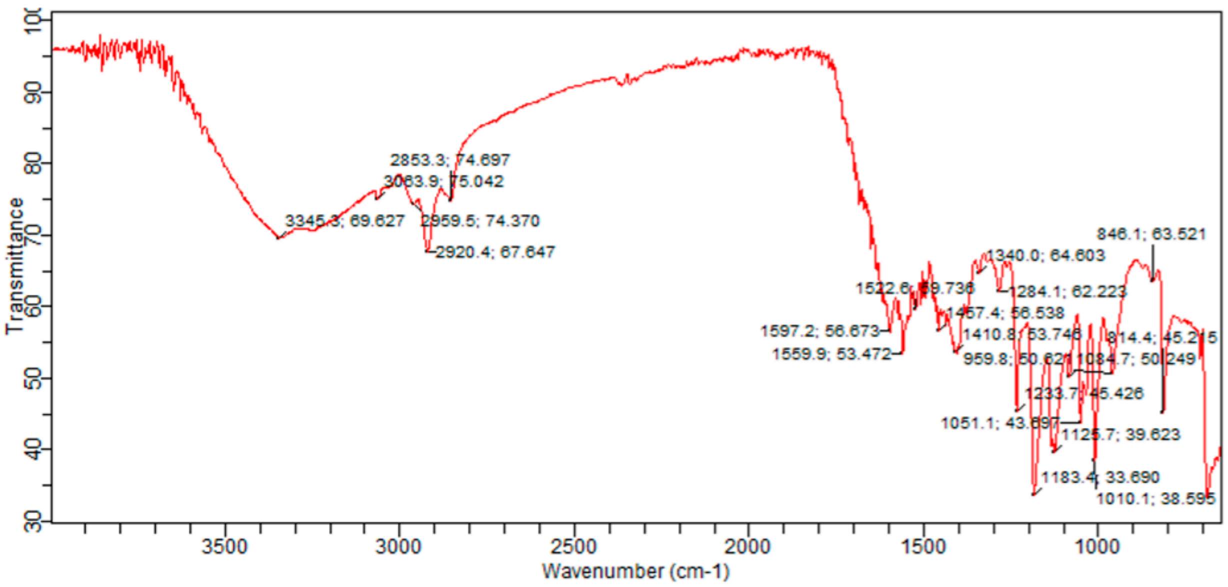

Figure S14-A Experimental FTIR spectrum of the compound 5b.

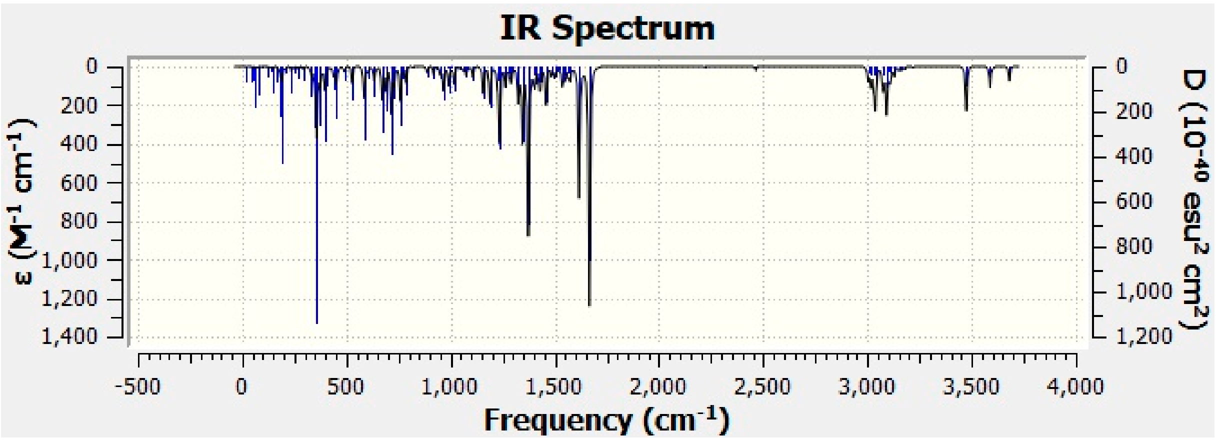

Figure S14-B Computed FTIR spectrum of the compound 5b

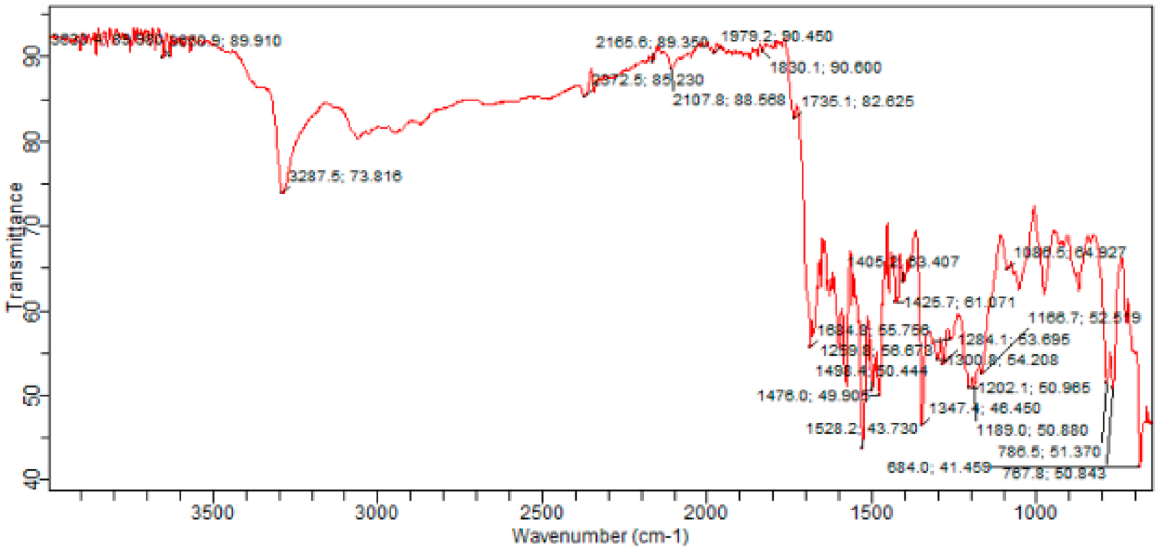

Figure S15-A Experimental FTIR spectrum of the compound 5c.

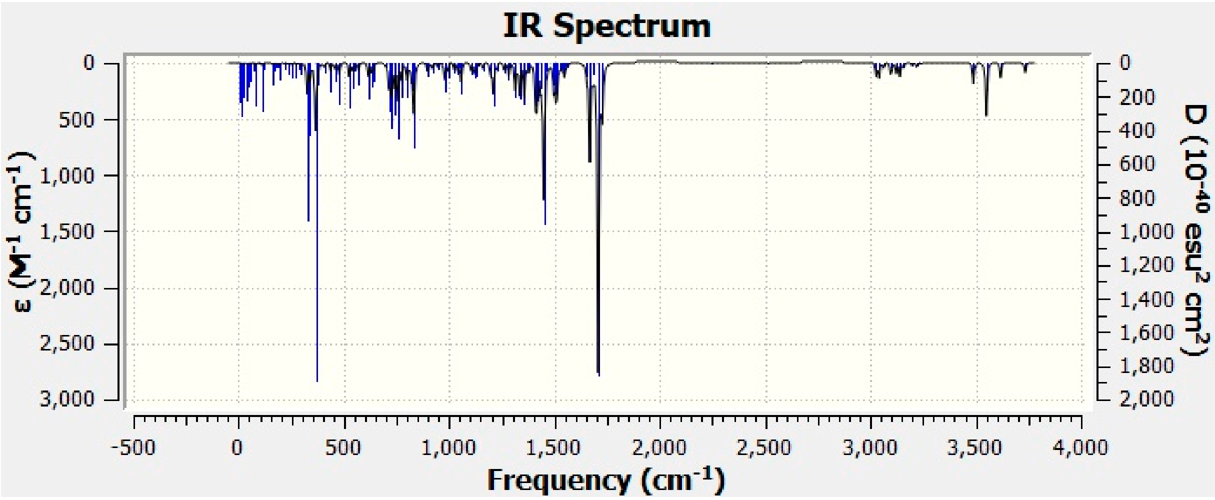

Figure S15-B Computed FTIR spectrum of the compound 5C

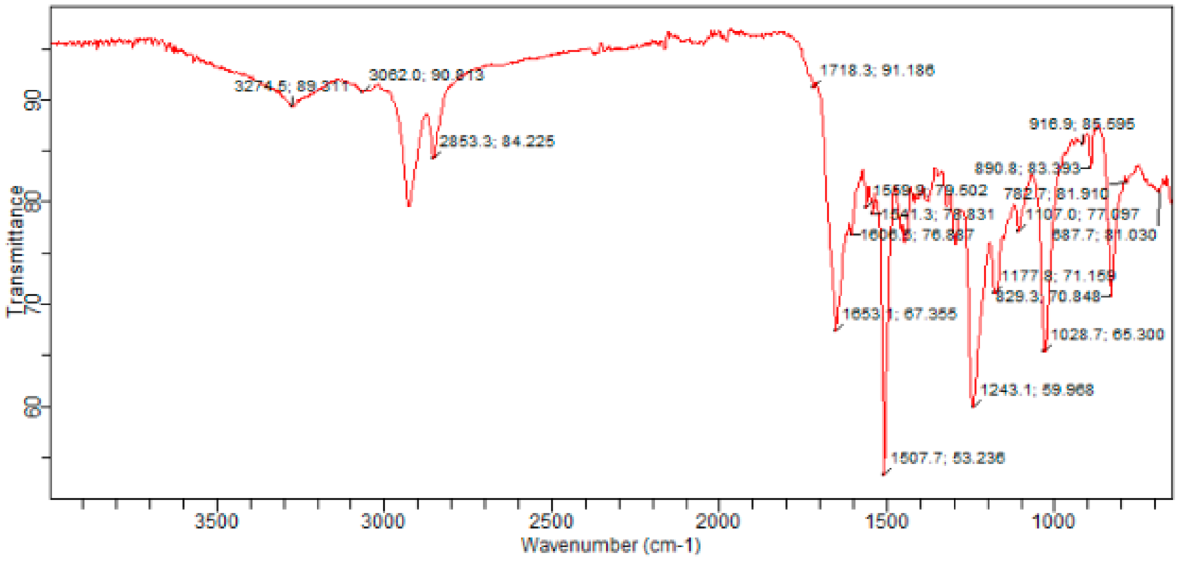

Figure S16-A Experimental FTIR spectrum of the compound 5d.

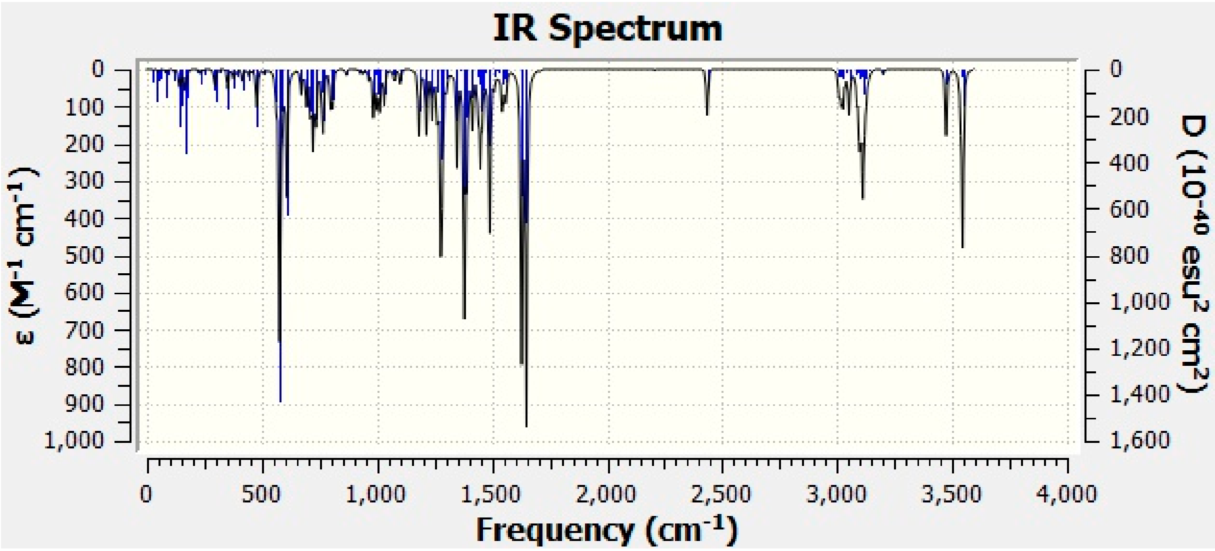

Figure S16-B Computed FTIR spectrum of the compound 5D

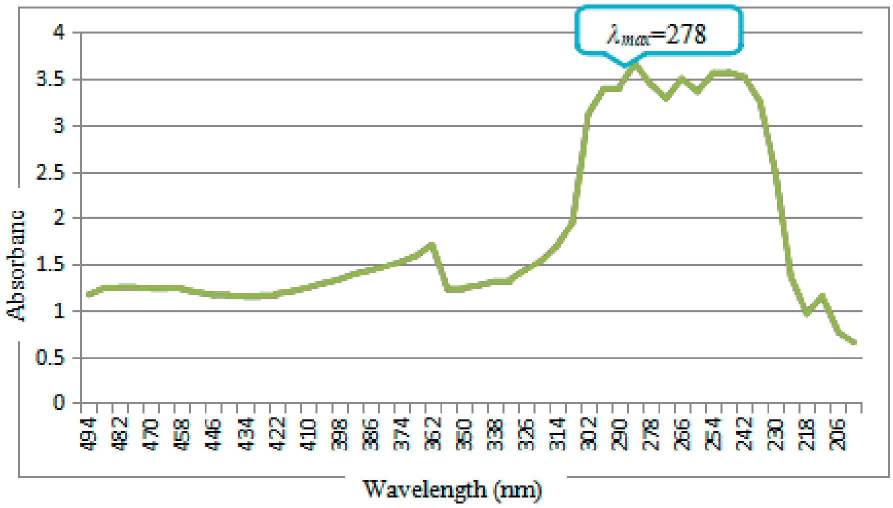

Figure S17 Experimental U.V **s**pectrum of the compound 5a.

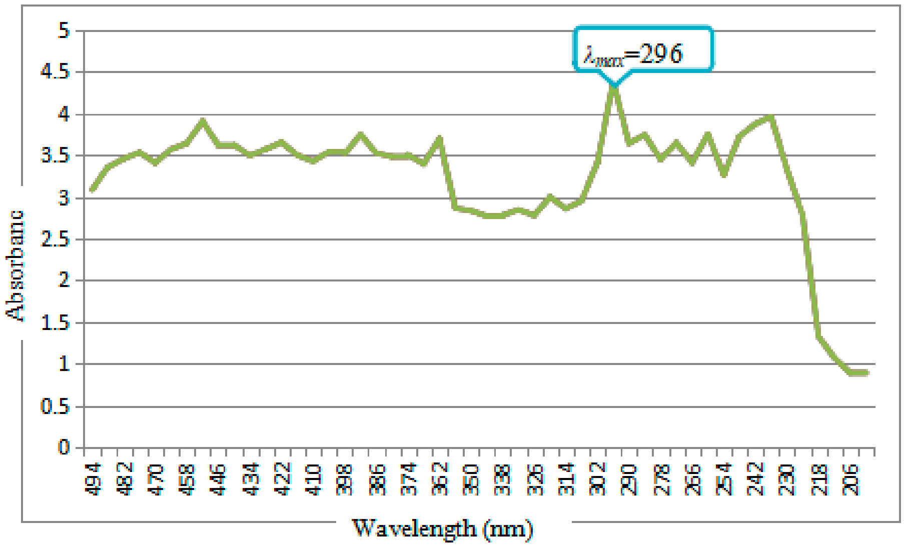

Figure S18 Experimental U.V spectrum of the compound 5b.

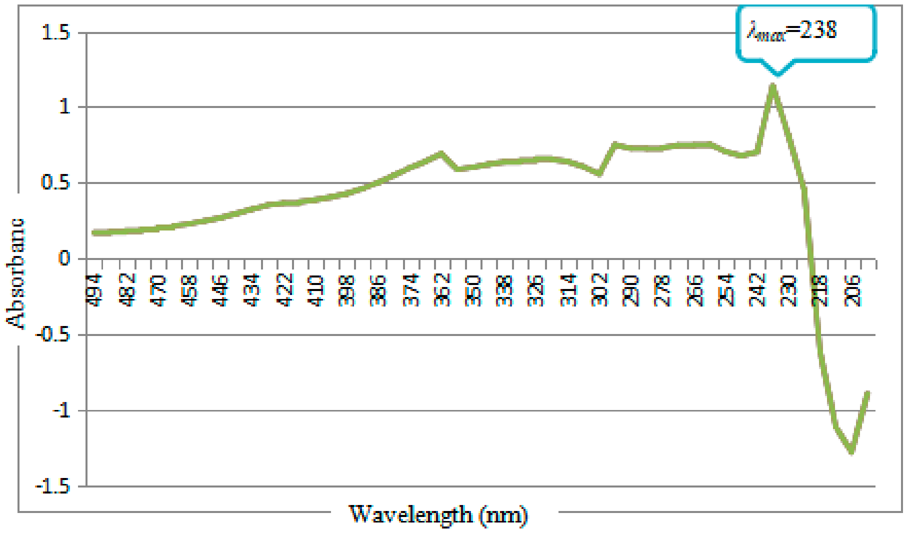

Figure S19 Experimental U.V spectrum of the compound 5c.

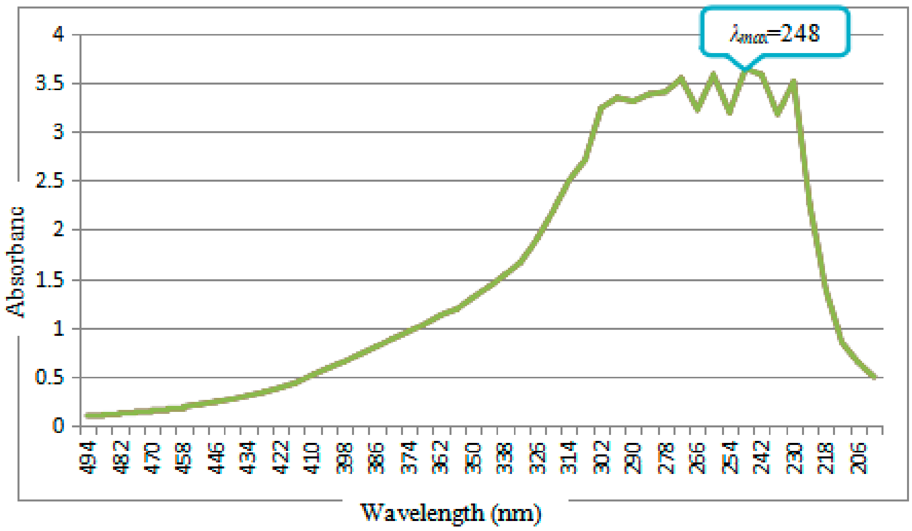

Figure S20 Experimental U.V spectrum of the compound 5d.

Table S1: Calculated FTIR analysis of compound 5a

Number	Frequency	Intensity	Mode
1	3654.82	14.8464	O1H, O23H τ
2	3557.69	32.1601	N18H, N42H τ
3	3522.53	22.6769	N14H, N39H τ
4	3432.28	62.7992	C22H, C45H τ
5	3105.83	8.5726	C12H, CH2 *Vas*
6	3057.38	43.2376	C9H, CH3, *Vas*
7	3090.99	12.1675	C8H, CH3 *Vas*
8	3078.59	24.5353	C2H, CH2 *Vas*
9	3045.72	42.1130	C8H, CH3 *Vas*
10	3036.31	12.5670	C20H, CH2 *Vas*
11	3035.14	12.0028	C6H, CH τ
12	3023.18	19.8087	C16H, CH2 *Vs*
13	2996.53	22.6798	C20H, CH2 *Vs*
14	2992.90	42.6684	C8, C9, C10, 3CH3 *Vs*
15	2991.45	32.8098	C2H, CH2 τ
16	2714.38	22.5746	S13H, τ, S-H stretching
17	1755.42	365.7537	C17O, C=O stretching
18	1715.32	302.7865	C11O, C=O stretching
19	1683.84	190.41	C3O, C=O stretching
20	1520.66	44.9011	N18H, τ
21	1516.12	18.7629	C16H, CH2 δ
22	1455.17	76.6507	C11N, C-N stretching
23	1429.64	22.7045	C2H, CH2, τ
24	1420.00	115.7500	C17N, C-N stretching
25	1404.88	190.1585	C17NC20, C-N-C stretching

26	1318.97	135.2218	C3-N, C-N stretching
27	1295.38	23.5220	C16h, CH2 ω
28	1277.37	11.7686	C2O, C-O stretching
29	1267.07	18.5832	C12H, CH2 ω
30	1261.98	16.9793	C7CH, C(CH3)3 ω
31	1230.63	78.5300	C7-C8, C-C stretching
32	1217.36	70.3951	C3N4, C-N stretching
33	1188.28	41.2599	C20N, C-N stretching
34	1180.06	19.0418	C16N, C-N stretching
35	1138.14	37.3946	C2C3, C-C stretching
36	1024.18	27.9167	C6N, C-N stretching
37	995.07	38.6202	C6C12, C-C stretching
38	972.77	15.9585	C2C3, C-C stretching
39	845.38	25.7074	C21-C22, C triple bond C stretching
40	723.67	55.8124	C6-C12, C-C stretching
41	701.91	33.4679	C2-C3, C-C stretching
42	652.02	60.5375	C6-C11, C-C stretching
43	644.47	33.2415	C16-C17, C-C stretching
44	469.16	144.1508	C7,C8,C9,C10, C(CH3)3

Table S2 Calculated FTIR value of compound 5b

Number	Frequency	Intensity	Mode
1	3632.67	17.4266	O1H, O19H
2	3539.52	22.0133	N12H, N32H
3	3431.26	62.9486	C16H, C35H
4	3124.25	23.7285	C8H, CH3

5	3104.97	32.9829	C17H, CH2
6	3074.40	26.3713	C7C9,2CH3 VS
7	3059.91	54.1406	C7C8C9,3CH3 VS
8	3058.02	4.1685	C10H, CH, C14H, CH2 VAS
9	3054.72	13.7518	C7H, CH3, C8H, CH3 VS
10	3050.71	10. 8969	C2H, CH2
11	3049.93	9.6759	C17H, CH2
12	3046.18	11.1094	C14H, CH2
13	3043.92	24.2654	C9CH3 ρ
14	3004.98	34.3718	C7C8C9, 3CH3 VS
15	2995.90	15.0812	C14H, CH2
16	2995.00	8.8383	C8C7,2CH3 ρ
17	2987.14	25.5880	C9, CH3 ρ
18	2717.94	7.0489	S18H, S38H
19	2232.86	0.1127	C15-C16H, C35H
20	1743.38	278.5018	C11O, C13O
21	1699.78	216.8776	C3O, C5O
22	1578.44	26.8887	C7C8C9,3CH3 ρ
23	1551.53	0.5677	C14H, CH2 δ
24	1550.83	6.1753	C7C8C9, 3CH3 ω
25	1547.52	2.9653	C7C8C9,3CH3 β
26	1540.89	11.5262	C7C8C9,3CH3 γ
27	1539.50	8.6424	C2H, CH2 γ
28	1530.78	28.9373	N12H, N32H

29	1524.67	9.9990	C17H, CH2 ρ
30	1490.40	25.8939	C7C8C9,3CH3 ρ
31	1470.93	17.8141	C10H, C31H
32	1461.22	14.7667	C7C8C9, 3CH3 γ
33	1450.05	16.7066	C7C9,2CH3
34	1440.99	39.9957	C2O, CH2 C1O, OH ω
36	1412.05	1.9429	C4H, CH2 τ
37	1401.38	0.7354	C2O, C10, CH2OH *VS*
38	1385.54	186.3270	C11N, C12N, *VAS*
39	1367.47	59.2692	C10, C17, CH2CH *VS*
40	1302.39	43.8108	C14H, CH2 *VAS*
41	1262.23	27.0330	C17, CH2 *VAS*
42	1240.26	55.4571	C6C7C8C9 C(CH3)3 τ
43	1184.40	14.8364	C14N, C12N *VAS*
44	1115.09	102.9399	C2O, C1O *VAS*
45	1075.25	26.3270	C8, CH3 τ
46	1027.25	25.5179	C14, CH2 τ
47	1003.03	18.6417	C2, CH2 *VAS*
48	975.01	11.9723	C14, C15
49	951.75	3.0256	C6C7, C6C8
50	922.69	1.6344	C6C8
51	892.40	11.3003	C6C9, CCH3
52	791.86	33.5934	N12H, N32H
53	787.10	47.6971	C16H, C35H
54	736.01	33.8550	S18H,S38H
55	432.37	30.3723	C15-C16

Table S3 Calculated FTIR value of compound 5c

Number	Frequency	Intensity	Mode
1	3658.21	13.3074	O1H, O27H
2	3538.37	31.2411	N17H, N40H
3	3476.89	79.3900	N13H, N37H
4	3411.68	58.4136	C9H, C33H
5	3148.94	12.1384	ArC23H, ArC44H *VS*
6	3131.61	10.4545	ArC24H, ArC45H *VS*
7	3128.21	6.4708	ArC21H, ArC43H
8	3113.48	6.8227	ArC25H, ArC46H
9	3103.99	0.3904	C7H, CH2 *Vas*
10	3080.74	1.3198	C11H, CH2 *Vas*
11	3053.38	14.0940	C15H, CH2 *Vas*
12	3049.50	20.0877	C2H, CH2 *Vs*
13	3037.58	24.1171	C11H, CH2 *Vs*
14	3016.92	17.5057	C19H, CH2 *Vas*
15	3008.88	19.1130	C6H, CH *Vs*
16	3005.80	32.5808	C2H, CH2 *Vs*
17	2995.83	20.7794	C7H, CH2 *Vs*
18	2994.24	25.1916	C15, CH2 *Vs*
19	2981.52	36.1651	C19H, CH2 *Vs*
20	2725.99	13.8807	S12H, SH, *Vs*
21	1758.57	191.9732	C16O, C18O τ
22	1741.10	584.9304	C10O, C14O τ
23	1713.70	160.9245	C3O, C5O τ
24	1702.49	14.7270	C8-C9, C=C stretching
25	1641.77	25.8638	Ar C-C=C stretching β
26	1625.99	38.9333	Ar C-C=C stretching γ
27	1565.34	4.1804	C19H, CH2 δ

28	1538.02	23.1231	Ar CH γ
29	1534.78	35.1298	C11, CH2 δ
30	1526.18	13.1360	C7, CH2 δ
31	1520.69	44.8041	N17H.N40H, *Vs*
32	1470.64	52.1169	C6H, C30H, *Vs*
33	1437.07	29.9094	C2O, CH2OH τ
34	1424.00	24.4720	C19H, CH2, γ
35	1417.75	257.41	C15-C16, C-C stretching
36	1395.04	83.8373	C6-C11 CH-CH2 stretching
37	1393.18	68.6335	C11H, CH2 τ
38	1365.88	122.0699	C11H, CH2 τ
39	1346.50	103.2814	C15H, CH2 τ
40	1309.61	72.4509	C6H, CH2 τ
41	1278.08	72.5647	C2H, CH2 δ
42	1275.85	32.8618	Ar-C19H, Ar-CH2 *Vs*
43	1228.47	100.3316	C2O, CH2OH, *Vas*
44	1199.33	37.2771	C7N, C4N, C-N stretching
45	1184.88	58.7560	C19N, C17N, C-N stretching
46	1120.36	51.8744	C2, CH2 γ
47	1095.20	50.6771	C11S, C-S stretching
48	977.61	39.2423	C2H, CH2 τ
49	833.22	59.9536	N13H, N37H τ
50	777.60	45.2154	C2-C3, C-C stretching
51	726.80	29.5022	N17H, N40H, *Vs*
52	688.04	39.2199	C9H, CH *Vs*
53	624.98	28.3184	N13H,N-H stretching

54	506.02	36.8205	C8-C9, C-C stretching
55	493.53	82.692	O1H, O-H stretching
56	443.87	13.1024	ArC22Cl, C-Cl stretching

Table S4 Calculated FTIR value of compound 5d

Number	Frequency	Intensity	Mode
1	3621.58	19.7808	O1H, O19H, τ
2	3508.70	11.6562	N13H, N29H τ
3	3430.27	51.7406	C9H, C25H τ
4	3086.99	51.1863	C16H, CH3 *Vas*
5	3080.42	20.2370	C17H, CH3, *Vas*
6	3066.40	36.0686	C11H, CH2 *Vs*
7	3058.72	54.8652	C18H, CH3 *Vas*
8	3051.48	14.8957	C6H, CH τ
9	3050.76	20.0080	C2H, CH2 *Vas*
10	3007.97	33.2339	C2H, CH2 *Vs*
11	2998.22	19.5429	C7H, CH2 *Vs*
12	2989.26	302683	C16H, CH3 *Vs*
13	2978.35	22.9678	C18H, CH3 *Vs*
14	2724.41	20.2735	S12H τ
15	1722.86	293.9397	C3O, C=O stretching
16	1700.08	189.3932	C10O, C=O stretching
17	1568.42	16.6033	C17H, CH3 *Vs*
18	1558.48	12.3824	C15C, C(CH3)3 γ
19	1540.50	19.2073	C11H, CH2 δ
20	1523.49	11.0404	N13H, N-H stretching
21	1516.31	13.0738	C7H, CH2 β

22	1468.93	133.4156	C6H, CH τ
23	1449.44	40.6060	C2H, CH2 τ
24	1426.48	184.4114	C6-C10, C-C stretching
25	1406.32	16.2139	C7H, CH2 τ
26	1370.53	59.9527	C11H, CH2 τ
27	1300.35	23.1666	C15N, C-N stretching
28	1283.24	127.5717	C7H, CH2 ω
29	1248.08	15.1662	C16H, CH3 τ
30	1234.88	89.7864	C2H, CH2 ω
31	1203.24	40.1332	C7N, C4N, C-N stretching
32	1109.37	51.0731	C2O, C-O stretching
33	1017.38	39.1014	C6N, C-N stretching
34	804.40	73.3019	N13H, N-H stretching
35	788.82-753.76	43.5659-57.5094	C9H, C25H τ
36	752.52	25.4420	C2-C3, C-C stretching
37	721.79	11.3691	S12H, S-H stretching
38	678.04	23.5267	C7N, C4N, C-N stretching
39	607.31	20.0016	C10O, C14O, C=O stretching
40	582.28	20.3127	N13H, N29H, N-H stretching
41	516.85	81.7906	C8-C9, C-triple bond C stretching
42	486.75	97.9254	O1H, O-H stretching
43	460.16	74.0266	C3O, C=O stretching
44	437.29	71.6609	C7N, C-N stretching

Table S5 Natural bond orbital (NBO) analysis of compound 5a

Number	Donor (i)	Types	Acceptor(i)	Types	E(2) kj/mol	E(j)E(i)(a.u)	F(i.j) (a.u)
1	C20-C21	σ	C21-C22	σ*	9.27	1.73	0.113
2	C22-H45	σ	C21-C22	σ*	7	1.62	0.095
3	C21-C22	σ	C20-C21	σ*	6.81	1.31	0.084
4	C17-O19	π	C17-O19	π*	1.33	0.36	0.021
5	C11-O15	π	C11-O15	π*	1.28	0.42	0.022
6	C3-O5	π	C3-O5	π*	0.92	0.39	0.018
7	C21-C22	π	N18-C20	σ*	5.08	0.63	0.051
8	C3-O5	π	O1-C2	σ*	3.37	0.66	0.042
9	C21-C22	π	C21-C22	σ*	3.07	0.67	0.041
10	C20-H43	σ	C21-C22	π*	5.4	0.63	0.052
11	C16-H40	σ	C17-O19	π*	4.37	0.52	0.046
12	C20-H44	σ	C21-C22	π*	4.12	0.63	0.046
13	O5	LP(2)	C3-N4	σ*	23.31	0.66	0.112
14	O19	LP(2)	C17-N18	σ*	23.05	0.69	0.114
15	O15	LP(2)	C11-N14	σ*	21.11	0.71	0.111
16	O15	LP(2)	C10-H34	σ*	0.51	0.68	0.017
17	N18	LP(1)	C17-O19	π*	69.24	0.26	0.119
18	N14	LP(1)	C11-O15	π*	59.01	0.29	0.117
19	N4	LP(1)	C3-O5	π*	57.65	0.26	0.109
20	N4	LP(1)	C11-O15	π*	1.4	0.28	0.018

Table S6 Natural bond orbital (NBO) analysis of compound 5b

Number	Donor (i)	Types	Acceptor(i)	Types	E(2) kj/mol	E(j)E(i)(a.u)	F(i.j) (a.u)
1	C14-C15	σ	C15-C16	σ*	9.32	1.73	0.113
2	C16-H35	σ	C15-C16	σ*	7.02	1.62	0.095
3	C15-C16	σ	C14-C15	σ*	6.82	1.31	0.085
4	C2-C3	σ	C2-H20	σ*	0.5	1.02	0.02
5	C11-O13	π	C11-O13	π*	1.05	0.43	0.02
6	C3-O5	π	C3-O5	π*	0.88	0.37	0.017
7	C15-C16	π	N12-C14	σ*	5.04	0.63	0.05
8	C15-C16	π	C14-H33	σ*	3.11	0.67	0.041
9	C11-O13	π	C11-O13	σ*	1.05	0.43	0.02
10	C3-O5	π	C3-O5	σ*	0.88	0.37	0.017
11	C10-H31	σ	C11-O13	π*	6.45	0.54	0.056
12	C2-H20	σ	C3-O5	π*	6.05	0.52	0.053
13	C14-H33	σ	C15-C16	π*	5.38	0.63	0.052
14	C14-H34	σ	C15-C16	π*	4.11	0.64	0.046
15	O5	LP(2)	C2-C31	σ*	9.5	0.58	0.096
16	N12	LP(1)	C14-H33	σ*	6.92	0.66	0.064
17	O1	LP(2)	C2-C3	σ*	6.87	0.64	0.06
18	N4	LP(1)	C10-C11	σ*	6.38	0.57	0.057
19	N4	LP(1)	C6-C7	σ*	5.81	0.61	0.057
20	O1	LP(2)	C2-H21	σ*	5.68	0.69	0.056
21	O5	LP(2)	C3-N42	σ*	5.37	0.65	0.116
22	N4	LP(1)	C10-C17	σ*	5.23	0.57	0.052
23	N12	LP(1)	C11-O134	π*	1.94	0.3	0.101
24	N4	LP(1)	C3-O55	π*	0.68	0.24	0.099

Table S7 Natural bond orbital (NBO) analysis of compound 5c

Number	Donor (i)	Types	Acceptor(i)	Types	E(2) kj/mol	E(j)E(i)(a.u)	F(i.j) (a.u)
1	C11-H35	σ	N4-C6	σ*	5.72	0.82	0.061
2	C9-H33	σ	C7-C8	σ*	5.59	0.96	0.066
3	C23-C24	σ	C22-Cl26	σ*	5.39	0.84	0.06
4	C20-C21	σ	C22-Cl26	σ*	5.03	0.83	0.058
5	C15-C16	σ	C15-H39	σ*	0.51	1.03	
6	C21-C22	π	C20-C251	π*	8.74	0.3	0.067
7	C20-C25	π	C21-C222	π*	2.09	0.26	0.069
8	C23-C24	π	C21-C222	π*	1.63	0.26	0.068
9	C16-O18	π	C16-O18	π*	1.46	0.36	0.022
10	C10-O14	π	C10-O14	π*	1.17	0.43	0.021
11	C3-O5	π	C3-O5	π*	0.95	0.36	0.018
12	C23-C24	π	C20-C252	π*	0.6	0.28	0.068
13	C20-C25	π	C23-C242	π*	0.39	0.28	0.068
14	C21-C22	π	C23-C242	π*	0.08	0.3	0.069
15	C20-C25	π	N17-C19	σ*	4.77	0.6	0.052
16	C8-C9	π	N4-C7	σ*	4.62	0.58	0.046
17	C3-O5	π	O1-C2	σ*	2.85	0.63	0.038
18	C8-C9	π	C7-H31	σ*	2.78	0.65	0.038
19	C20-C25	π	C19-H41	σ*	2.27	0.63	0.037
20	C8-C9	π	C7-H32	σ*	1.65	0.67	0.03
21	C8-C9	π	C7-H32	σ*	1.09	0.66	0.024
22	C7-H31	σ	C8-C9	π*	4.72	0.59	0.047
23	C15-H39	σ	C16-O18	π*	4.48	0.53	0.047
24	C6-H30	σ	C10-O14	π*	4.34	0.55	0.046
25	C15-H38	σ	C16-O18	π*	4.2	0.53	0.045
26	C2-H29	σ	C3-O5	π*	3.69	0.52	0.042
27	N13-C15	σ	C10-O14	π*	0.8	0.79	0.024

28	O5	LP(2)	C2-C31	σ*	8.8	0.59	0.095
29	O1	LP(2)	C2-C3	σ*	7.96	0.66	0.065
30	N17	LP(1)	C19-H42	σ*	7.62	0.63	0.067
31	N4	LP(1)	C6-C10	σ*	7.56	0.59	0.063
32	N13	LP(1)	C15-H38	σ*	6.57	0.62	0.061
33	N13	LP(1)	C15-H39	σ*	5.45	0.62	0.056
34	O5	LP(2)	C3-N42	σ*	5.22	0.66	0.117
35	N4	LP(1)	C7-H31	σ*	5.05	0.63	0.054
36	C26	LP(2)	C23-C24	σ*	0.5	0.87	0.019
37	N13	LP(1)	C10-O144	π*	9.53	0.29	0.108
38	N17	LP(1)	C16-O186	π*	8.66	0.26	0.119
39	C26	LP(3)	C21-C221	π*	4.62	0.32	0.066
40	N4	LP(1)	C3-O56	π*	1.8	0.26	0.113
41	O1	LP(2)	C3-O5	π*	1.4	0.32	0.02
42	N4	LP(1)	C8-C9	π*	1.16	0.32	0.019

Table S8 Natural bond orbital (NBO) analysis of compound 5d

Number	Donor (i)	Types	Acceptor(i)	Types	E(2) kj/mol	E(j)E(i)(a.u)	F(i.j) (a.u)
1	C7-C8	σ	C8-C9	σ*	8.89	1.72	0.11
2	C9-H25	σ	C8-C9	σ*	7.35	1.62	0.097
3	C9-H25	σ	C7-C8	σ*	6.58	1.02	0.073
4	C8-C9	σ	C7-C8	σ*	6.38	1.31	0.082
5	N13-H29	σ	C6-C10	σ*	5.88	0.96	0.068
6	C17-H34	σ	N13-C15	σ*	5.3	0.83	0.06
7	C11-H27	σ	N4-C6	σ*	5.25	0.82	0.059
8	N4-C7	σ	C6-H22	σ*	0.53	1.13	0.022
9	C10-O14	π	C10-O14	π*	1.38	0.59	0.027
10	C3-O5	π	C3-O5	π*	0.91	0.36	0.017
11	C8-C9	π	N4-C7	σ*	5.09	0.62	0.05
12	C3-O5	π	O1-C2	σ*	2.97	0.63	0.039

13	C8-C9	π	C7-H23	σ*	2.94	0.68	0.04
14	C8-C9	π	C7-H24	σ*	1.93	0.69	0.033
15	C10-O14	π	C10-N13	σ*	0.51	0.95	0.02
16	C7-H23	σ	C8-C9	π*	5.27	0.62	0.051
17	C7-H24	σ	C8-C9	π*	4.5	0.63	0.047
18	C2-H20	σ	C3-O5	π*	3.11	0.52	0.038
19	C6-C11	σ	C10-O14	π*	2.76	0.74	0.043
20	N4-C7	σ	C8-C9	π*	2.08	0.84	0.037
21	C7-H23	σ	C8-C9	π*	0.94	0.62	0.022
22	O5	LP(2)	C2-C31	σ*	8.8	0.59	0.095
23	O1	LP(2)	C2-C3	σ*	7.96	0.66	0.065
24	N17	LP(1)	C19-H42	σ*	7.96	0.66	0.065
25	N4	LP(1)	C6-C10	σ*	7.62	0.63	0.067
26	N13	LP(1)	C15-H38	σ*	7.56	0.59	0.063
27	N17	LP(1)	C19-H42	σ*	6.57	0.62	0.061
28	N13	LP(1)	C15-H39	σ*	5.45	0.62	0.056
29	O5	LP(2)	C3-N42	σ*	5.22	0.66	0.117
30	N4	LP(1)	C7-H31	σ*	5.05	0.63	0.054
31	C26	LP(2)	C23-C24	σ*	0.5	0.87	0.019
32	N4	LP(1)	C3-O5	π*	58.98	0.26	0.111
33	N13	LP(1)	C10-O14	π*	40.22	0.35	0.108
34	O1	LP(2)	C3-O5	π*	1.45	0.31	0.02
35	N4	LP(1)	C8-C9	π*	0.94	0.36	0.018
36	O14	LP(2)	C3-O5	π*	0.57	0.25	0.011
